# Supplementary material for: B-Cell Lymphoma-Extra Large Promotes Gastric Tumorigenesis via Apoptosis-Independent Wnt–β-Catenin Activation and Peroxisome Proliferator–Activated Receptor γ Suppression
Source: Cell Mol Gastroenterol Hepatol. 2026 Sep 8;21(1):101880. doi: 10.1016/j.jcmgh.2026.101880 (PMC13685607; doi:10.1016/j.jcmgh.2026.101880)

A

Cleaved Caspase-3

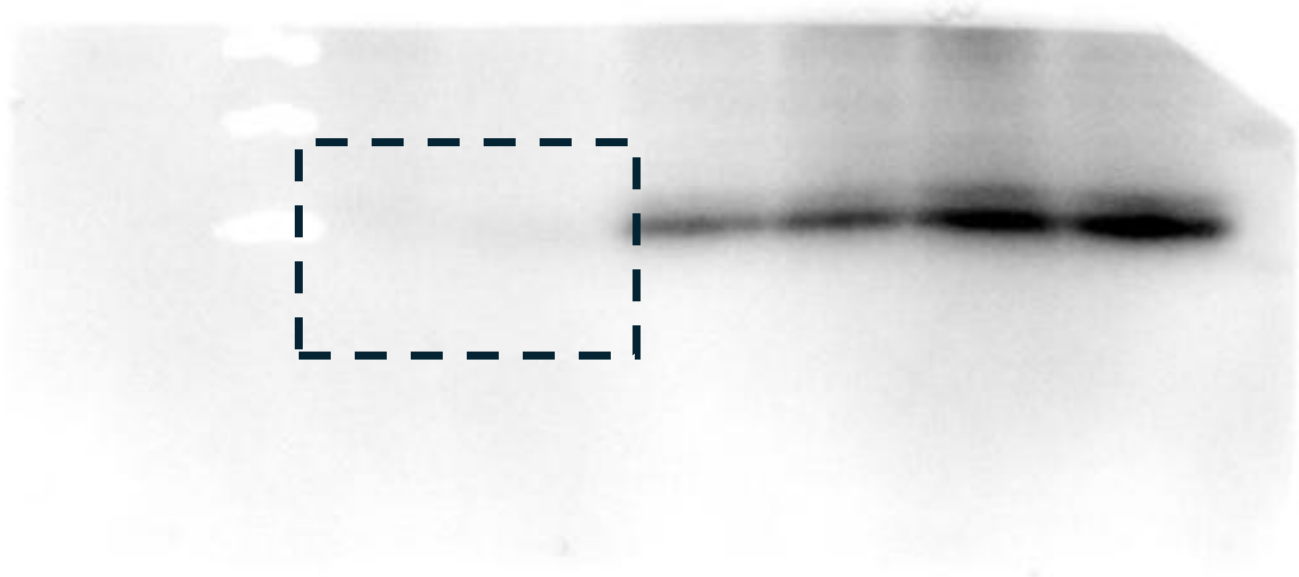

B

B catenin

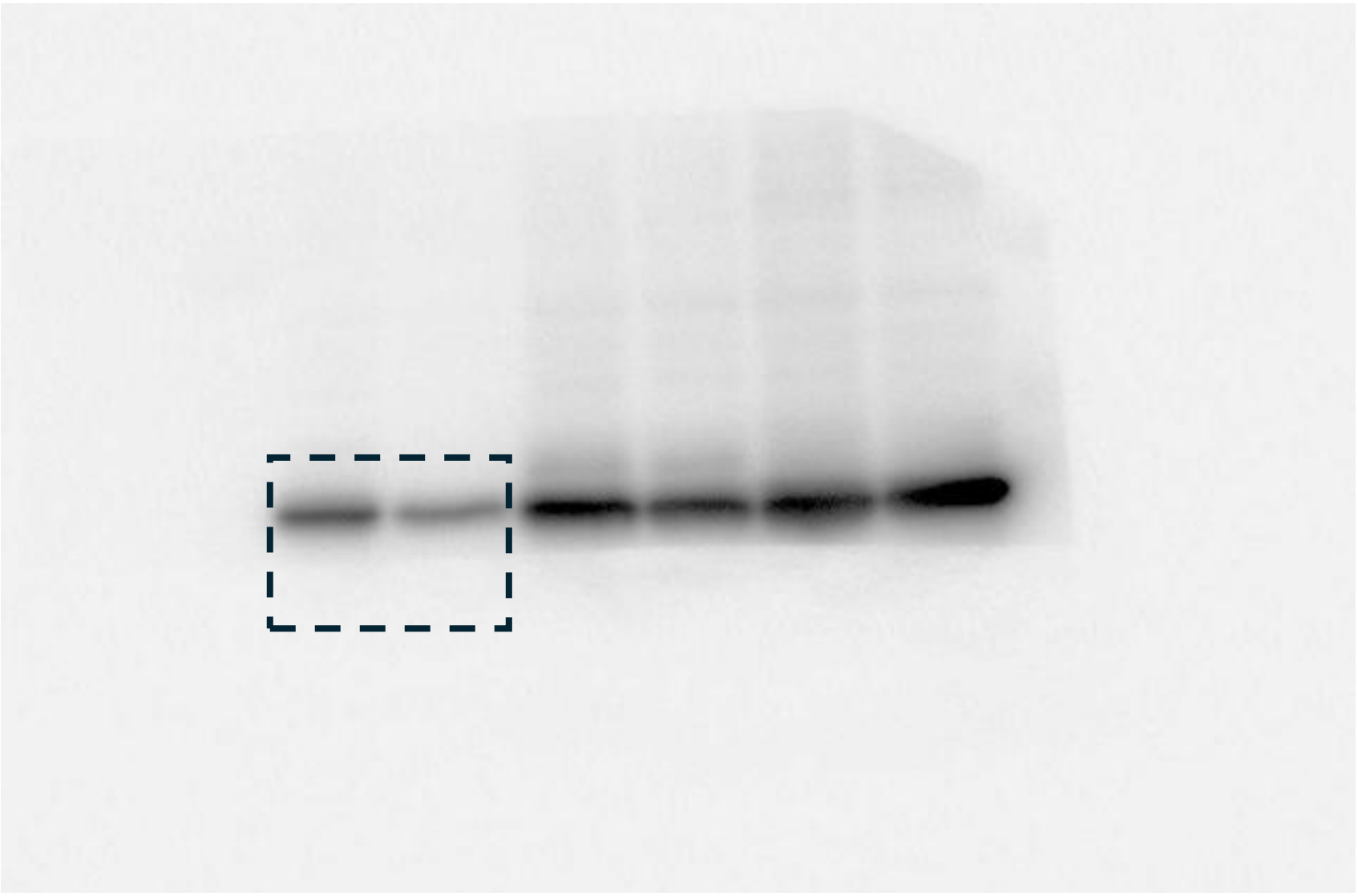

C

Caspase-3

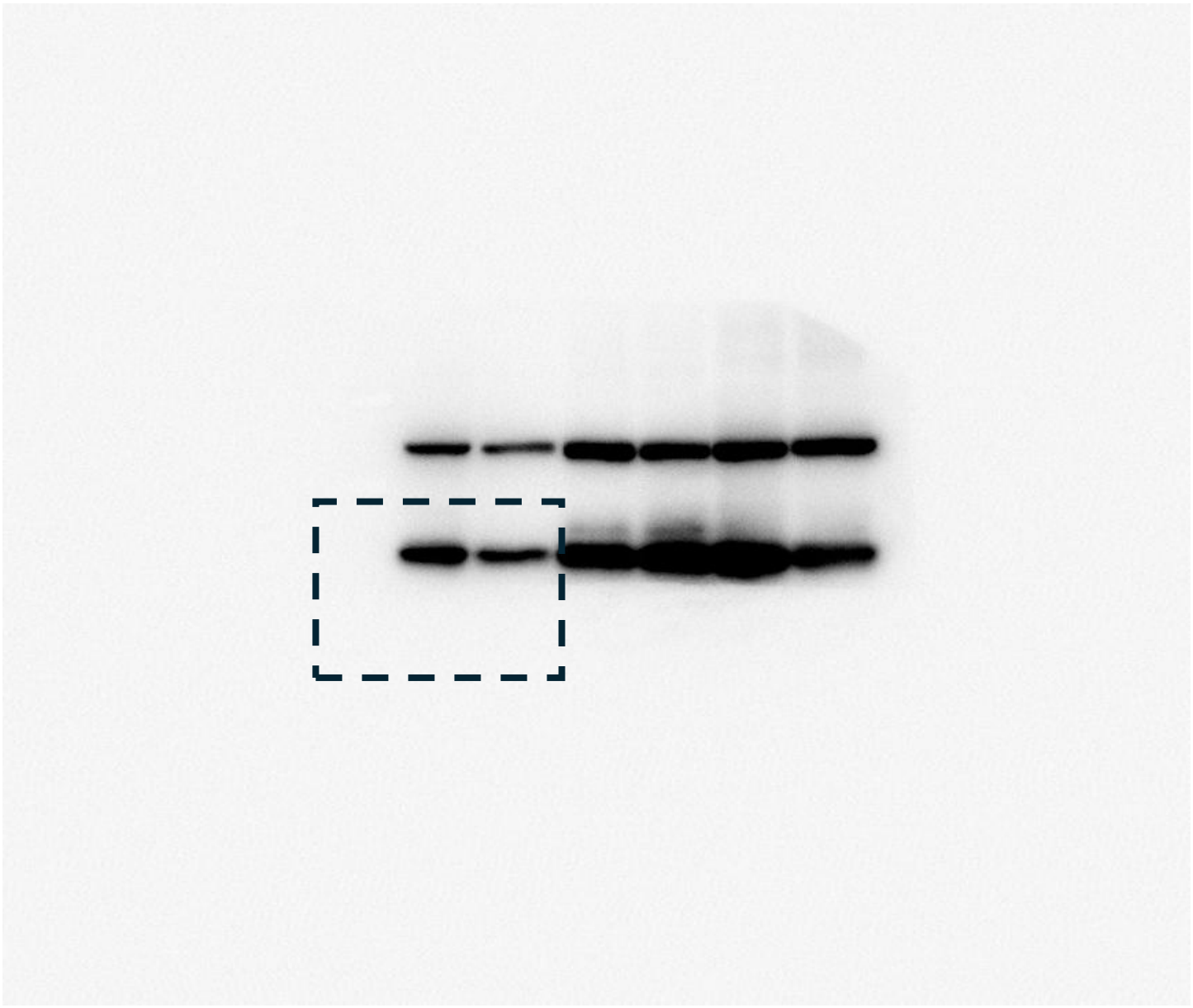

D

Bcl-xL

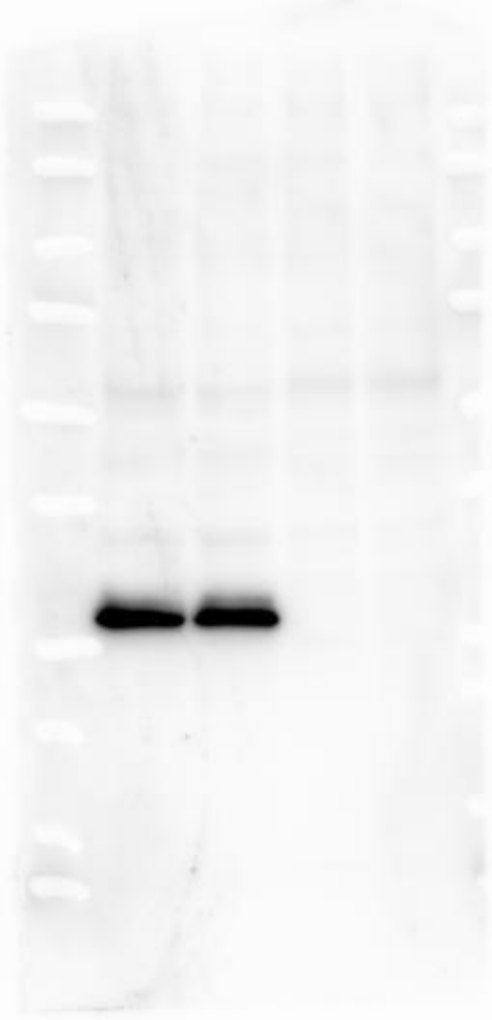

E

Cyclin D1

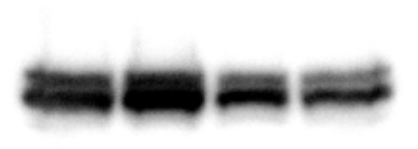

F

β-actin

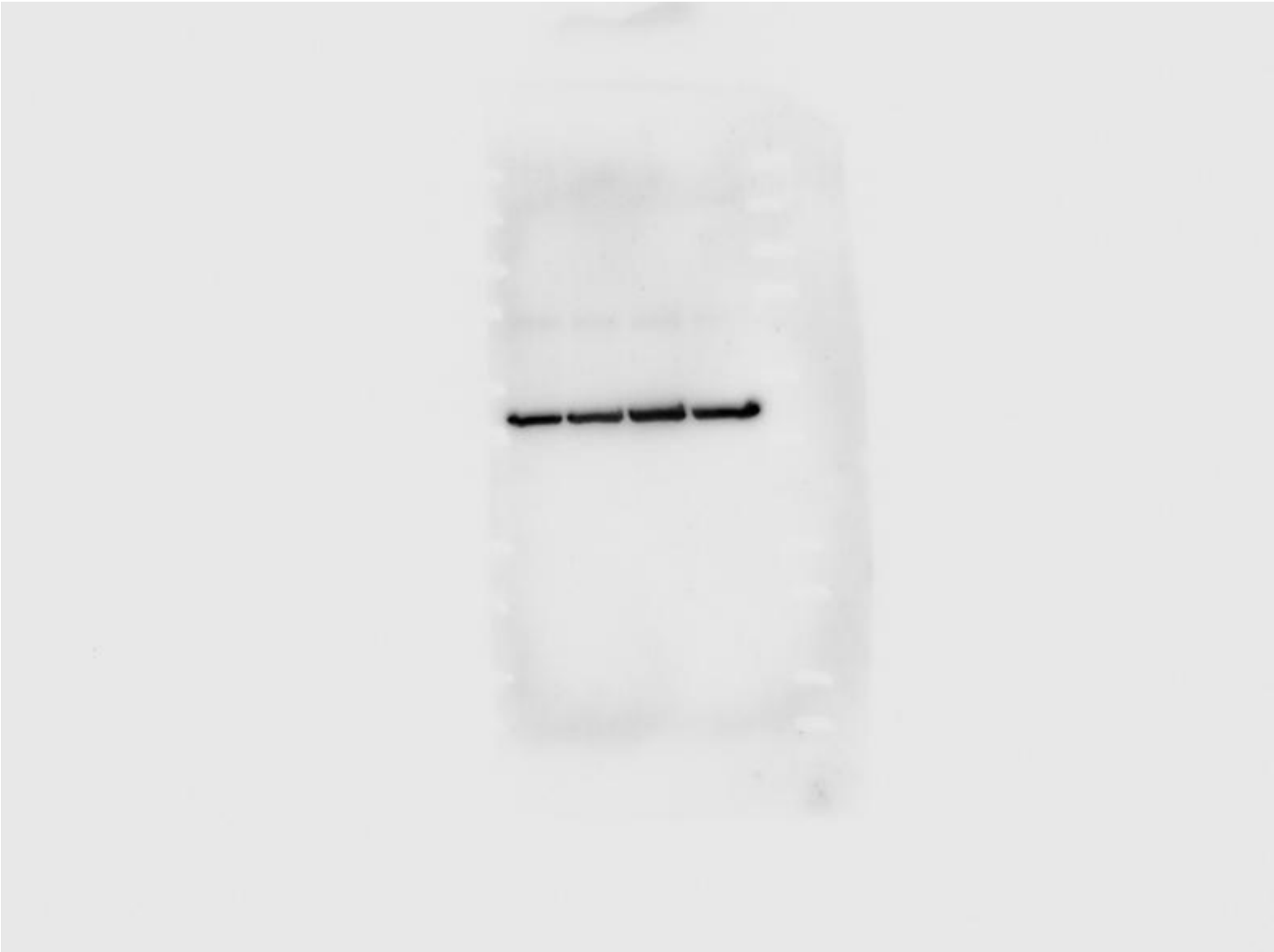

G

PPARγ

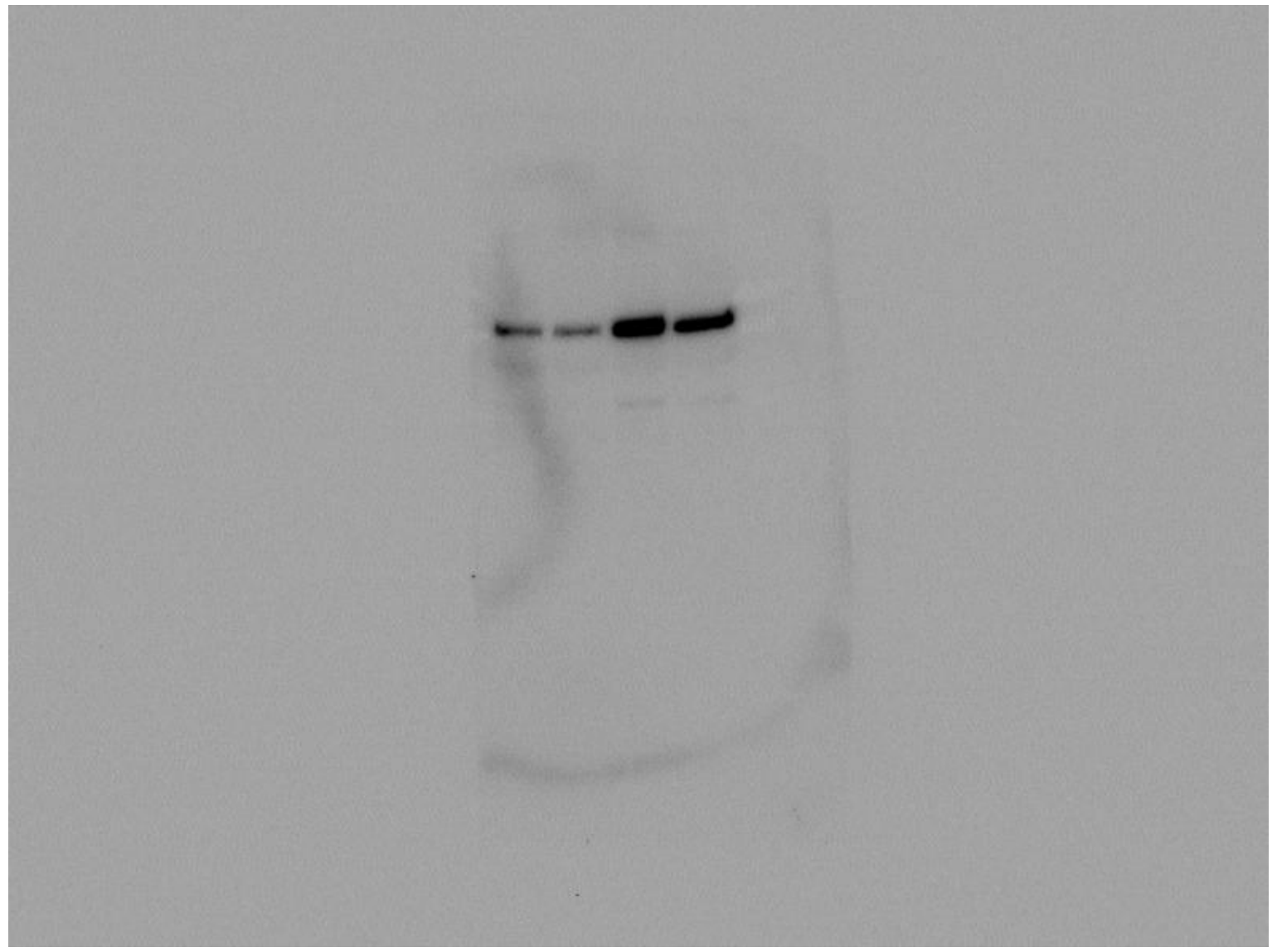

Supplement: Supplementary file 1 [file mmc1.pdf]
