# Supplementary material for: B-Cell Lymphoma-Extra Large Promotes Gastric Tumorigenesis via Apoptosis-Independent Wnt–β-Catenin Activation and Peroxisome Proliferator–Activated Receptor γ Suppression
Source: Cell Mol Gastroenterol Hepatol. 2026 Sep 8;21(1):101880. doi: 10.1016/j.jcmgh.2026.101880 (PMC13685607; doi:10.1016/j.jcmgh.2026.101880)

cmgh

CELLULAR AND MOLECULAR GASTROENTEROLOGY AND HEPATOLOGY

ORIGINAL RESEARCH

B-Cell Lymphoma-Extra Large Promotes Gastric Tumorigenesis via Apoptosis-Independent Wnt–β-Catenin Activation and Peroxisome Proliferator–Activated Receptor γ Suppression

Yukiko Oya,[1,*] Junya Arai,[1,2,3,*] Yoku Hayakawa,[1] Toshiro Shiokawa,[1] Shintaro Shinohara,[1] Masahiro Hata,[1] Yuki Matsushita,[1] Hiroto Kinoshita,[1] Chie Uekura,[1] Ken Kurokawa,[1] Sohei Abe,[1] Mayo Tsuboi,[1] Sozaburo Ihara,[1] Nobumi Suzuki,[1] Hayato Hikita,[4] Tetsuo Takehara,[4] Timothy C. Wang,[3] and Mitsuhiro Fujishiro[1]

[1]Department of Gastroenterology, Graduate School of Medicine, The University of Tokyo, Tokyo, Japan; [2]Division of Gastroenterology, The Institute of Medical Science, Asahi Life Foundation, Tokyo, Japan; [3]Division of Digestive and Liver Disease, Department of Medicine, Columbia University, New York, New York; and [4]Department of Gastroenterology, Graduate School of Medicine, The University of Osaka, Osaka, Japan

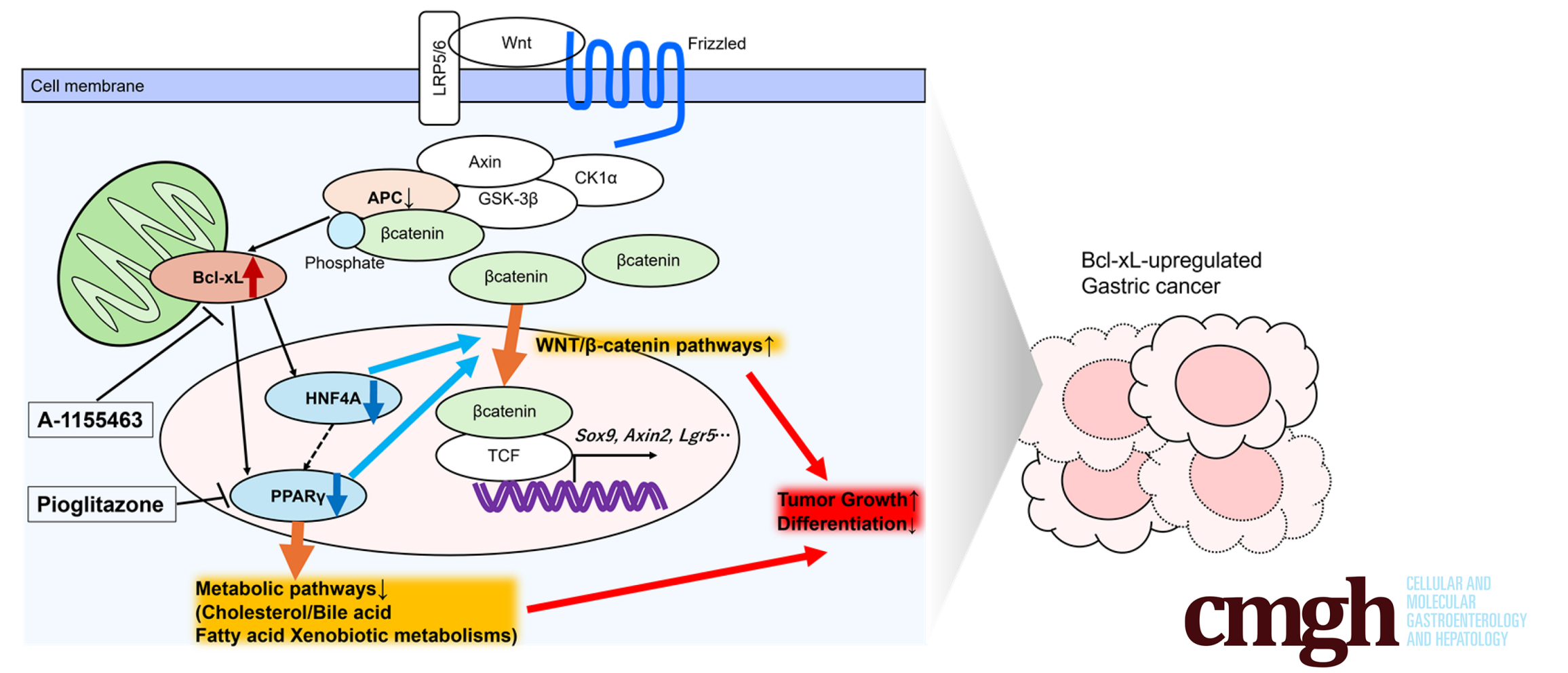

SUMMARY

B-cell lymphoma–extra-large promotes gastric tumorigenesis by enhancing Wnt–β-catenin signaling and suppressing peroxisome proliferator–activated receptor γ–associated metabolic programs. Genetic or pharmacologic targeting of this pathway may inhibit gastric tumor growth, highlighting B-cell lymphoma–extra-large as a potential therapeutic target and peroxisome proliferator–activated receptor γ activation as a possible protective strategy.

WHAT YOU NEED TO KNOW

Background: B-cell lymphoma–extra-large is frequently upregulated in gastric cancer, but its functional role beyond apoptosis, particularly in regulating Wnt signaling and tumor metabolism, remains poorly understood.

Impact: This study demonstrates that B-cell lymphoma–extra-large promotes gastric tumorigenesis by enhancing Wnt–β-catenin signaling through suppression of hepatocyte nuclear factor 4 α and peroxisome proliferator–activated receptor γ, thereby sustaining proliferation and impairing metabolic differentiation.

Future Directions: Targeting B-cell lymphoma–extra-large–mediated Wnt activation or restoring peroxisome proliferator–activated receptor γ signaling, including pharmacologic peroxisome proliferator–activated receptor γ agonists, may represent novel preventive or therapeutic strategies for gastric cancer.

BACKGROUND & AIMS: The Wnt–β-catenin and Yes-associated protein pathways cooperatively drive gastrointestinal tumor progression. BCL2L1, a Yes-associated protein target gene, encodes the anti-apoptotic isoform B-cell lymphoma–extra-large, which is highly expressed in multiple cancers, but its role in gastric cancer remains unclear.

METHODS: We generated *Mist1*-CreERT; *Apc*$^{flox/flox}$ and *bcl-xl*$^{flox/flox}$ mice to study gastric tumorigenesis with or without B-cell lymphoma–extra-large. Gastric organoids and xenografts were used to assess proliferation and downstream signaling, and findings were validated in human gastric cancer tissues and databases.

RESULTS: Deletion of B-cell lymphoma–extra-large significantly reduced tumor growth and proliferation in *Apc*-deficient tumors, organoids, and xenografts. Nuclear β-catenin and Wnt target gene expression were attenuated, partly through derepression of *Hnf4a and Pparg*, indicating that B-cell lymphoma–extra-large promotes Wnt signaling. Concurrently, downregulation of *Hnf4a* and *Pparg* contributes to the inhibition of multiple metabolic pathways. In human gastric cancer, high *BCL2L1* expression correlated with elevated Wnt target genes and poor prognosis, whereas *PPARG* expression inversely correlated with *BCL2L1* and associated with favorable outcomes. Epidemiologic analysis suggested that pioglitazone, a peroxisome proliferator–activated receptor γ agonist, reduced gastric cancer incidence in diabetic patients.

CONCLUSIONS: B-cell lymphoma–extra-large promotes gastric tumorigenesis through dual mechanisms—enhancing Wnt signaling and suppressing peroxisome proliferator–activated receptor γ—and highlight B-cell lymphoma–extra-large as a potential therapeutic target while suggesting peroxisome proliferator–activated receptor γ activation may be protective. *(Cell Mol Gastroenterol Hepatol 2027;21:101880; https://doi.org/10.1016/j.jcmgh.2026.101880)*

Keywords: Bcl-xL; Gastric Cancer; PPARγ; Wnt–β-catenin.

Gastric cancer (GC) is one of the most common cancers in the world. In recent years, the number of deaths due to GC has been declining due to a decrease in the *Helicobacter pylori* infection rate and improved health screening.[1] However, the 5-year survival rate for advanced GC remains low, and the 5-year survival rate for GC with distant metastasis is less than 10%.[2] Although several new drugs including immune checkpoint inhibitors and targeted antibodies have been applied to GC treatment and found to be effective in disease control, most advanced GCs become resistant to these drugs after several cycles and hardly show complete response. Thus, there is a demand for seeking additional therapeutic targets to control GCs that are resistant to currently available therapies.[3–8]

In gastric glands, stem and progenitor cells in the isthmus continuously supply differentiated cells and maintain mucosal homeostasis. Many gastric stem cell markers have been reported, such as LGR5, CCK2R, AXIN2, FZD7, CD44, SOX2, and MIST1 for antrum and SOX9, IQGAP3, STMN1, SOX2 and MIST1 for corpus.[9–17] Induction of genetic mutations in stem cells is sufficient to generate gastric tumors, whereas that in differentiated cells fails to develop tumors.[13,18,19] For example, deletion of *Apc* in the gastric stem cells results in the formation of tumors in the gastric antrum.[20–23] On the other hand, GC only develops in the presence of multiple mutations or a combination of mutation and additional chemical stimuli.[22,24,25] In gastric carcinogenesis, mutated stem cells are supported by the surrounding microenvironment. Many cell types are involved in such microenvironment, including inflammatory cells, fibroblasts, nerves, vascular endothelial cells, luminal bacteria, hormones, and secreted mucins, and these stromal components promote tumor growth by releasing a variety of molecules that directly activate cancer cell growth signals or restructure the surrounding area.[12,26–30] Among them, the Wnt–β-catenin signaling pathway has been identified to play a central role in the development and progression of GC.[21,31–33]

We previously reported that the Wnt pathway and the Yes-associated protein (YAP) pathway are activated in GC cells through nerve-regulatory niche signals. YAP activation leads to upregulation of certain genes including *BCL2L1* and others, but the significance and role of these gene changes remain unknown.[23] *BCL2L1* is a gene encoding the Bcl2 family proteins B-cell lymphoma–extra-large (Bcl-xL) and B-cell lymphoma–extra-small (Bcl-xS), which regulate apoptosis by modulating mitochondrial membrane permeability; Bcl-xL is an inhibitor of apoptosis, whereas Bcl-xS is a promoter.[34–36] Bcl-xL is reported to be involved in other biological reactions, such as autophagy,[37,38] neural network formation,[39] and cellular metabolism.[37]

Bcl-xL is overexpressed in several cancers, and it has been reported that inhibition of Bcl-xL suppresses tumor growth. In pancreatic cancer, Bcl-xL was found to increase the incidence of pancreatic cancer by suppressing oncogene-induced senescence and apoptosis.[40] In colorectal and hepatocellular cancers, inhibition of Bcl-xL induced apoptosis, suggesting that combined therapies targeting Bcl-xL may increase the

*Authors share co-first authorship.

Abbreviations used in this paper: APC, adenomatous polyposis coli; AQP5, aquaporin-5; Bcl-xL, B-cell lymphoma–extra-large; Bcl-xS, B-cell lymphoma–extra-small; BSA, bovine serum albumin; cDNA, complementary DNA; DAB, 3,3'-diaminobenzidine; DMEM, Dulbecco's Modified Eagle Medium; GC, gastric cancer; GFP, green fluorescent protein; GSEA, Gene Set Enrichment Analysis; HNF4A, hepatocyte nuclear factor 4 α; LGR5, leucine-rich repeat-containing G protein-coupled receptor 5; M-A, *Mist1-CreERT; Apc*$^{flox/flox}$; M-AB, *Mist1-CreERT; Apc*$^{flox/flox}$; *bcl-xl*$^{flox/flox}$; M-B, *Mist1*-CreERT; *bcl-xl*$^{flox/flox}$; OE, overexpression; OR, odds ratio; PBS, phosphate-buffered saline; PCA, principal component analysis; PPARγ, peroxisome proliferator–activated receptor γ; qPCR, quantitative polymerase chain reaction; RT-PCR, real-time polymerase chain reaction; SOX9, SRY-box transcription factor 9; TAM, tamoxifen; TBS-T, Tris Buffered Saline with Tween 20; TCGA, The Cancer Genome Atlas; TROP2, trophoblast cell surface antigen 2; WT, wild-type; YAP, Yes-associated protein.

Most current article

© 2026 The Author(s). Published by Elsevier Inc. on behalf of American Gastroenterological Association Institute. This is an open access article under the CC BY license (http://creativecommons.org/licenses/by/4.0/).
2352-345X
https://doi.org/10.1016/j.jcmgh.2026.101880

efficacy of chemotherapy.[41–43] Overexpression of Bcl-xL and its tumor promoting role have been also reported in GCs.[44–46] However, the precise mechanism of how Bcl-xL is upregulated and promotes tumor growth has not been fully elucidated. In this study, we analyzed the function of Bcl-xL in GC and explored its potential as a new therapeutic target.

Results

B-Cell Lymphoma–Extra-Large Enhances the Gastric Tumorigenesis in Part Through Activation of Wnt Pathway

To investigate the role of Bcl-xL in gastric mucosa and tumorigenesis, we analyzed tissues from *Mist1*-CreERT; *bcl-xl*$^{flox/flox}$ (M-B), *Mist1*-CreERT; *Apc*$^{flox/flox}$ (M-A), *Mist1*-CreERT; *Apc*$^{flox/flox}$; *bcl-xl*$^{flox/flox}$ (M-AB), and *Mist1*-CreERT control (wild-type [WT]) mice. All mouse lines were generated on an LSL-TdTomato reporter background (Figure 1*A* and *B*). To characterize the expression pattern of Bcl-xL in normal and neoplastic gastric tissues, we performed fluorescent immunostaining for Bcl-xL on antral sections from each genotype. In M-B mice, Bcl-xL expression was markedly diminished in approximately 49% of glandular units relative to WT controls, indicating efficient Cre-mediated recombination in nearly one-half of the glands (Figure 1*C*). We next assessed the impact of Bcl-xL deficiency on tumor initiation and progression. Consistent with previous reports,[10] M-A mice developed distal antral tumors within 1 month of tamoxifen (TAM) administration. At 2 months post-TAM, we compared gastric tumor sizes between M-A and M-AB mice and found that tumors in M-AB mice were significantly smaller than those in M-A mice (Figure 1*D*). In detail, all mice in the M-A cohort developed large tumors that covered the entire antral region and appeared as conglomerates of multiple nodular lesions. This morphology suggests that these tumors may have arisen from the fusion of multiple independent tumor foci. In contrast, mice in the M-AB cohort developed only a single, relatively small tumor per mouse. The consistent presence of a single tumor in the M-AB group, without evidence of multiple tumor formation, is likely attributable to the tumor suppressive effect of Bcl-xL deletion. Histopathologic analysis also confirmed this reduction in size, although both tumors were classified as intramucosal tumors. In contrast, M-B mice, which harbored Bcl-xL deletion alone, showed no histologic abnormalities compared with WT controls after TAM treatment (Figure 1*E*).

Red fluorescent protein immunostaining confirmed robust TdTomato expression in both normal and tumor tissues (Figure 1*F*), indicating efficient Cre-mediated recombination. In M-B mice, sustained TdTomato fluorescence observed 2 months after TAM administration suggested that Bcl-xL ablation did not induce apoptosis and that Bcl-xL–deficient cells were able to survive long term. Quantitative real-time polymerase chain reaction (RT-PCR) using gastric tissue RNA further confirmed that Bcl-xL expression was decreased in M-B and M-AB mice, whereas it was elevated in M-A mice (Figure 1*G*).

To assess epithelial proliferative activity, we stained gastric tissues for Ki67. In both WT and M-B mice, Ki67-positive cells remained confined to the normal proliferative zones of the glands, indicating that loss of Bcl-xL alone does not disrupt baseline epithelial proliferation (Figure 2*A*). In contrast, M-A and M-AB mice exhibited distinct proliferation patterns in tumor regions: Ki67 expression was significantly higher in M-A tumors than in M-AB tumors (Figure 2*B*). These results indicate that the tumor suppressive effect of Bcl-xL loss is associated with a reduction in tumor cell proliferation. Notably, in M-A mice, immunohistochemical analyses using β-catenin and Ki67 and tdTomato and Ki67 costaining revealed an increase in Ki67^{+} cells not only among nuclear β-catenin^{+} cells and tdTomato^{+} cells but also in non–nuclear β-catenin cells and tdTomato^{-} cells. These findings suggest that hyperproliferation is not restricted to adenomatous polyposis coli (APC)-deficient cells but also extends to neighboring epithelial cells, supporting the presence of non–cell-autonomous proliferative effects induced by APC loss (Figure 2*C* and *D*).

We next investigated the impact of Bcl-xL on tumorigenesis, with a particular focus on the Wnt signaling pathway, given its well-established role in GC progression.[21,31–33] In WT mice, β-catenin localization was restricted to the plasma membrane, with no clear evidence of nuclear translocation (Figure 2*E*). In contrast, nuclear translocation of β-catenin was observed in the tumor regions of M-A and M-AB mice, consistent with *Apc* deletion (Figure 2*F*). Moreover, M-A mice exhibited strong Bcl-xL expression throughout the tumor tissue, with particularly high levels in certain tumor cells. The Bcl-xL positivity was associated with nuclear β-catenin. In M-AB mice, Bcl-xL expression was markedly reduced in tumor regions, except for a small fraction where expression persisted, indicating efficient Bcl-xL knockout in the tumor (Figure 2*F*). Bcl-xL knockout also slowed down the expansion of nuclear β-catenin clones over time (Figure 2*G*).

Regarding SRY-box transcription factor 9 (SOX9) expression, WT and M-B mice exhibited limited expression at the basal regions of the glandular ducts. In both M-A and M-AB mice, SOX9 expression was upregulated in tumor areas, but the number of SOX9-positive cells was significantly increased in M-A mice compared with that in M-AB mice (Figure 3*A* and *B*). Most cells showing nuclear β-catenin are also positive for SOX9 (Figure 3*C*). M-A mice also exhibited a significantly greater number of *Lgr5*-positive cells, compared with M-AB mice (Figure 3*D*). We next generated *Mist1*-CreERT; *Apc*$^{flox/flox}$; *Lgr5*-DTR-green fluorescent protein (GFP) mice and examined the correlation among leucine-rich repeat-containing G protein-coupled receptor 5 (LGR5)-GFP positivity, Bcl-xL positivity, and nuclear β-catenin localization. The results showed that LGR5 expression was associated with both Bcl-xL positivity and nuclear β-catenin accumulation (Figure 3*E* and *F*). These results suggest that Bcl-xL deletion leads to a partial attenuation of Wnt pathway activation.

To assess the role of Bcl-xL in metaplastic and dysplastic progression, we performed immunostaining for aquaporin-5 (AQP5) and trophoblast cell surface antigen 2 (TROP2). In

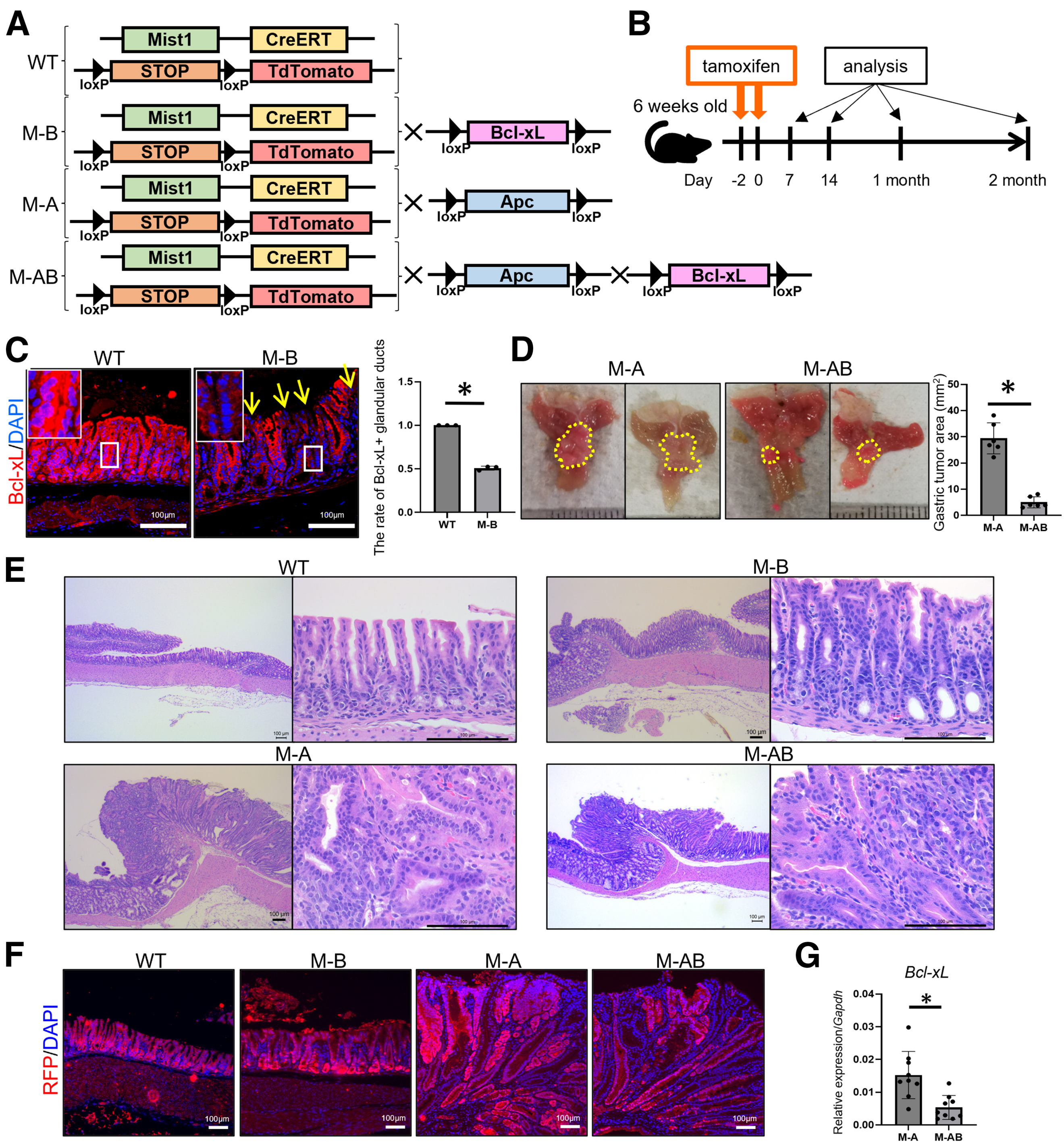

Figure 1. The deletion of Bcl-xL reduces tumor burden in *Apc*-deficient tumors. (*A*) Gene construction schema in each mouse model (WT, M-B, M-A, and M-AB mice). (*B*) The protocol schema of TAM administration. (*C*) Bcl-xL staining in WT and M-B mice. *Yellow arrows* indicate the glands without Bcl-xL expression. The rates of the Bcl-xL+ cells are quantified (n = 3 mice/group). The *P* value was calculated using a *t* test. (*D*) Macroscopic findings of gastric tumors in M-A and M-AB mice. Tumor area is surrounded by a *yellow dotted frame*. The tumor diameters are quantified (n = 6 mice/group). The *P* value was calculated using a *t* test. (*E*) Hematoxylin and eosin (H&E) staining of WT, M-B, M-A, and M-AB mice at antrum (representative images for n = 3 mice/group). (*F*) Red fluorescent protein (RFP) staining in WT, M-B, M-A, and M-AB mice (representative images for n = 3 mice/group). (*G*) RT-PCR of *Bcl2l1* (Bcl-xL) expression in WT, M-B, M-A, and M-AB mice (n = 9/group). The *P* value was calculated using a *t* test. Scale bars, 100 μm. Mean ± standard error of the mean. **P* < .05.

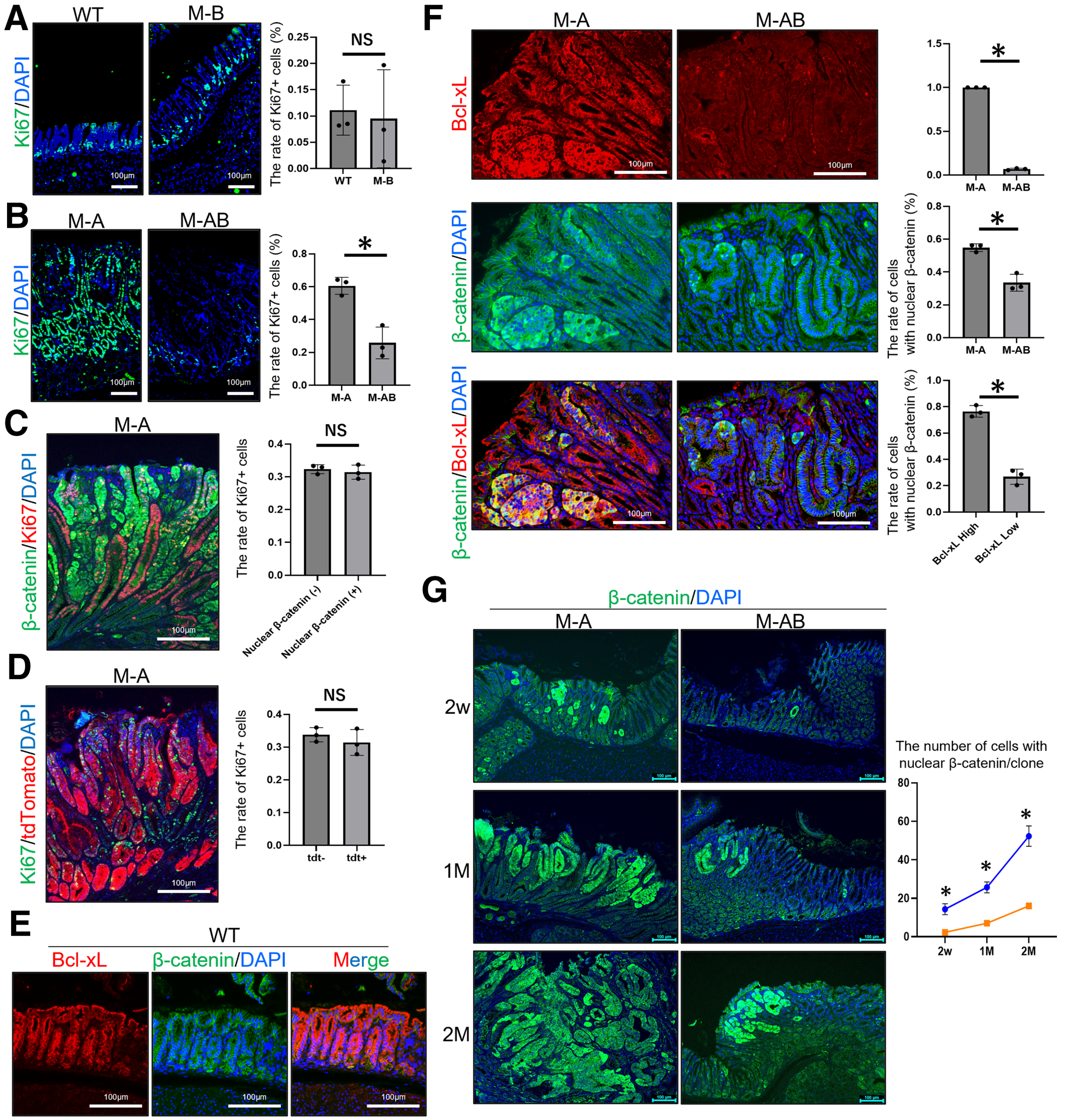

Figure 2. The deletion of Bcl-xL reduces tumor burden in *Apc*-deficient tumors. (*A*) Ki67 (*green*) staining in WT and M-B mice. The rates of the Ki67+ cells are quantified (n = 3/group). The *P* value was calculated using a *t* test. (*B*) Ki67 staining in M-A and M-AB mice. The rates of the Ki67+ cells are quantified (n = 3 mice/group). The *P* value was calculated using a *t* test. (*C*) β-catenin (*green*) and Ki67+ (*red*) staining in M-A mice. The rates of the Ki67+ cells are quantified, comparing non-nuclear and nuclear β-catenin cells (n = 3 mice/group). The *P* value was calculated using a *t* test. (*D*) tdTOMATO (*red*) and Ki67+ (*green*) staining in M-A mice. The rates of the Ki67+ cells are quantified, comparing tdTOMATO-positive and -negative cells (n = 3 mice/group). The *P* value was calculated using a *t* test. (*E*) Bcl-xL (*red*) and β-catenin (*green*) staining in WT mice (representative images for n = 3 mice/group). (*F*) Bcl-xL (*red*) and β-catenin (*green*) staining in M-A and M-AB mice. The rates of Bcl-xL+ cells and β-catenin–nuclear–translocated cells were quantified (n = 3 mice per group). In M-A mice, the rates of β-catenin–nuclear-translocated cells were quantified, comparing Bcl-xL-low and -high expressing cells (n = 3 mice per group). The *P* value was calculated using a *t* test. (*G*) β-catenin (*green*) staining in M-A and M-AB mice 2 weeks, 1 month, and 2 months after tamoxifen induction. The average number of β-catenin–nuclear-translocated cells per one clone were quantified (n = 10 clones per mouse, n = 3 mice per group). The *P* value was calculated using a *t* test. Scale bars, 100 μm. Mean ± standard error of the mean. *$P < .05$. DAPI, 4′,6-diamidino-2-phenylindole.

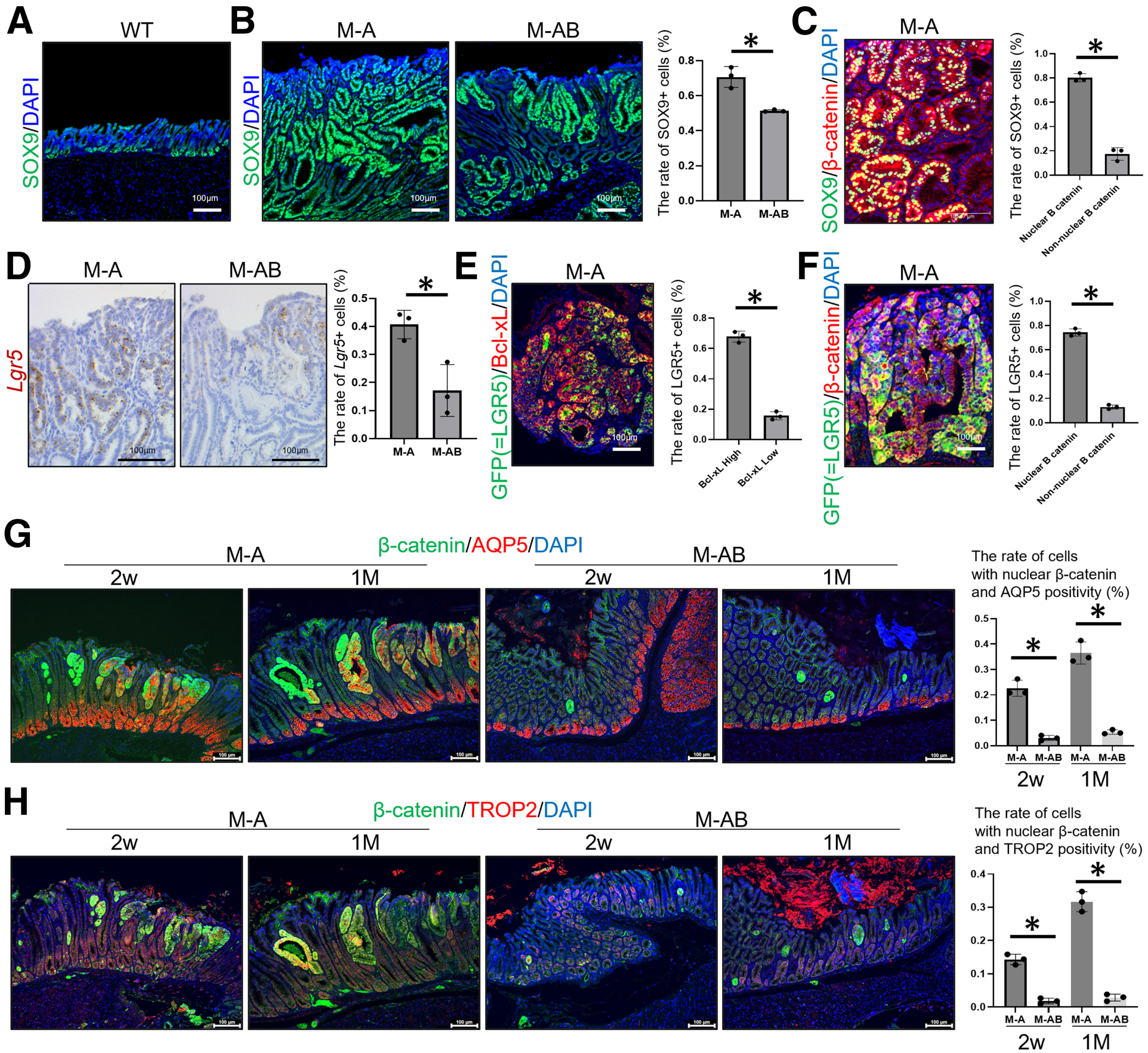

Figure 3. The deletion of Bcl-xL induces the inactivation of the Wnt–β-catenin pathway. (*A*) SOX9 staining in WT mice (representative images for n = 3 mice/group). (*B*) SOX9 staining in M-A and M-AB mice. The rates of the SOX9+ cells are quantified (n = 3 mice/group). The *P* value was calculated using a *t* test. (*C*) SOX9 (*green*) and β-catenin (*green*) staining in M-A mice. The rates of the SOX9+ cells are quantified, comparing non-nuclear and nuclear β-catenin cells (n = 3 mice/group). The *P* value was calculated using a *t* test. (*D*) RNA–in situ hybridization (ISH) using *Lgr5* probe in M-A and M-AB mice. The ratio of the *Lgr5*+ cells are quantified (n = 3 mice/group). The *P* value was calculated using a *t* test. (*E*) GFP (*green*) and Bcl-xL (*red*) staining in *Mist1*-creERT; *Apc* $^{flox/flox}$; *Lgr5*-GFP-DTR mice. The rates of GFP (=LGR5)-positive cells were quantified, comparing Bcl-xL-low and -high expressing cells (n = 3 mice per group). The *P* value was calculated using a *t* test. (*F*) GFP (*green*) and β-catenin (*red*) staining in *Mist1*-creERT; *Apc* $^{flox/flox}$; *Lgr5*-GFP-DTR mice. The rates of GFP (=LGR5)-positive cells were quantified, comparing non-nuclear and nuclear β-catenin cells (n = 3 mice/group). The *P* value was calculated using a *t* test. (*G*) β-catenin (*green*) and AQP5 (*red*) staining in M-A and M-AB mice 2 weeks and 1 month after TAM induction. The rates of nuclear β-catenin cells with AQP5 positivity are quantified (n = 3 mice/group). The *P* value was calculated using a *t* test. (*H*) β-catenin (*green*) and TROP2 (*red*) staining in M-A and M-AB mice 2 weeks and 1 month after TAM induction. The rates of nuclear β-catenin cells with TROP2 positivity are quantified (n = 3 mice/group). The *P* value was calculated using a *t* test. Scale bars, 100 μm. Mean ± standard error of the mean. *$P < .05$. DAPI, 4′,6-diamidino-2-phenylindole.

M-A mice, most cells with nuclear β-catenin were positive for both AQP5 and TROP2. In contrast, M-AB mice exhibited a marked reduction in AQP5- and TROP2-positive cells, accompanied by a decrease in nuclear β-catenin, suggesting a delay in metaplastic and dysplastic transformation compared with M-A mice over time. These findings indicate that Bcl-xL promotes metaplastic and dysplastic progression via activation of the WNT pathway (Figure 3*G* and *H*).

To directly assess apoptosis, we performed immunostaining for cleaved caspase-3, as Bcl-xL is known to exert

anti-apoptotic effects. However, no cleaved caspase-3–positive cells were detected in the gastric tissues of WT, M-B, M-A, or M-AB mice (Figure 4A). Apoptosis was also assessed at early time points in M-B and M-AB mice following TAM administration. No clear evidence of apoptosis in epithelial cells was observed from day 7 (before tumor formation) to one month (after tumor formation) after TAM treatment (Figure 4B). Thus, although Bcl-xL is upregulated in *Apc*-deficient gastric tumors, its anti-apoptotic function is limited in this tumor model. Given that Bcl-xL is a transcriptional target of YAP,[23] we also analyzed YAP expression. In WT and M-B mice, YAP expression was restricted to the base of the glandular epithelium. In contrast, both M-A and M-AB mice exhibited strong YAP expression in tumor regions; however, no significant difference was observed between these groups, suggesting that Bcl-xL deletion does not influence YAP pathway activation (Figure 4C).

To further evaluate gene expression changes, quantitative RT-PCR was performed using complementary DNA (cDNA) synthesized from gastric tissues of M-A and M-AB mice. Wnt target genes, including *Lgr5, Axin2, Sox9, Fzd7,* and *Ccnd1*, were markedly downregulated in M-AB mice, compared with M-A mice. In contrast, *Lgr4* and *cMyc* expression did not differ significantly between M-A and M-AB mice (Figure 4D). Collectively, these results indicate that Bcl-xL promotes gastric tumorigenesis in part through enhancing Wnt activation, thereby contributing to tumor progression, although these differences may also reflect the smaller tumor burden and lower number of APC-deficient cells in M-AB mice.

B-Cell Lymphoma–Extra-Large Activates the Wnt–β-Catenin Pathway in Tumor Organoids and Xenografts

To investigate the role of Bcl-xL in established gastric tumors in vitro, we employed a gastric organoid culture system. Organoids were derived from the normal gastric antrum of *Apc*$^{flox/flox}$ and WT mice, infected with Cre-expressing lentivirus, and selected with puromycin to generate genetically modified organoids (Figure 5A). Initially, we aimed to generate organoids from *bcl-xl*$^{flox/flox}$ and *Apc*$^{flox/flox}$; *bcl-xl*$^{flox/flox}$ mice. However, we were unable to establish stable cultures. In contrast, *Apc*-deficient organoids were successfully generated. Thus, WT and *Apc*-deficient organoids (hereafter referred to as Apc organoids) were used for further analysis.

To assess the effect of Bcl-xL on cell proliferation, we treated both WT and Apc organoids with the Bcl-xL–specific inhibitor (A-1155463) and monitored changes in organoid size. In WT organoids, Bcl-xL inhibition did not affect growth and apoptosis (Figure 5B–D and Supplementary Western blotting A–C). In contrast, Apc organoids exhibited a dose-dependent reduction in size upon Bcl-xL inhibition, indicating that Bcl-xL promotes proliferation in the *Apc*-deficient context (Figure 5C). There was no significant difference in organoid growth between WT and *Apc*-deficient organoids.

To further evaluate Bcl-xL function, we generated complex mutant organoids using LSL-*Kras*^{G12D}; LSL-*Trp53*^{R172H}; *Apc*$^{flox/flox}$ and LSL-*Kras*^{G12D}; LSL-*Trp53*^{R172H}; *Apc*$^{flox/flox}$; *bcl-xl*$^{flox/flox}$ mice infected with Cre-lentivirus and selected (hereafter referred to as KPA and KPAB organoids, respectively) (Figure 6A and B). We selected the additional *Kras*^{G12D} and *Trp53*^{R172H} mutations in the *Apc*-mutant GC model based on previous studies,[47,48] to enable the establishment of stable, genetically modified gastric organoids for functional analysis. Fluorescent immunostaining, Western blotting, and quantitative polymerase chain reaction (qPCR) confirmed Bcl-xL deletion in KPAB organoids (Figure 6B–E).

Western blot analysis also showed reduced expression of Cyclin D1 in KPAB organoids (Figure 3D and Supplementary Western blotting D–F). β-catenin nuclear localization was markedly reduced in KPAB organoids compared with KPA organoids (Figure 6E). qPCR showed that Wnt target genes *Axin2* and *Ccnd1* were downregulated in KPAB organoids, while no significant changes were detected in *Lgr4 or cMyc* (Figure 6F).

To evaluate the impact of Bcl-xL on proliferation, we compared organoid growth between KPA and KPAB under different culture conditions. In standard medium, no significant difference in growth was observed (Figure 6G). However, under Wnt-deficient conditions (Dulbecco's Modified Eagle Medium [DMEM]/F12 supplemented only with B27 and N2), KPAB organoids showed significantly smaller size than KPA organoids by day 5 (Figure 6H). Furthermore, treatment of KPA organoids with Bcl-xL inhibitor reduced organoid size, whereas KPAB organoids were unaffected by the inhibitor (Figure 6I). These findings indicate that Bcl-xL is dispensable for proliferation under optimal growth conditions but becomes critical for sustaining tumor growth in APC-deficient organoids under growth factor–limited conditions, which more closely reflect the in vivo tumor environment. Finally, we confirmed that there was no difference in apoptosis occurrence between KPA and KPAB organoids (Figure 6J).

Next, to assess the in vivo tumorigenic potential of Bcl-xL-deficient cells,[49] KPA and KPAB organoids were subcutaneously injected into nude mice, and tumor growth was monitored. KPAB-derived tumors were significantly smaller than KPA-derived tumors on both day 14 and day 21 (Figure 7A), indicating that loss of Bcl-xL again impairs xenografted, *Apc*-deleted tumor growth.

To further investigate molecular changes, xenograft tumors were harvested 1 month after injection and analyzed by immunohistochemical staining. GFP-positive cells, indicating organoid-derived tumor cells, were identified in both KPA and KPAB xenografts. In KPAB tumors, Bcl-xL expression was markedly reduced in GFP-positive cells, confirming successful knockout (Figure 7B). Nuclear localization of β-catenin was also decreased in KPAB tumors (Figure 7C), along with reduced expression of Wnt target genes Cyclin D1 and SOX9 and a proliferation marker Ki67, whereas no significant differences in YAP expression was observed between KPA and KPAB tumors, consistent with findings from the genetically engineered mouse and

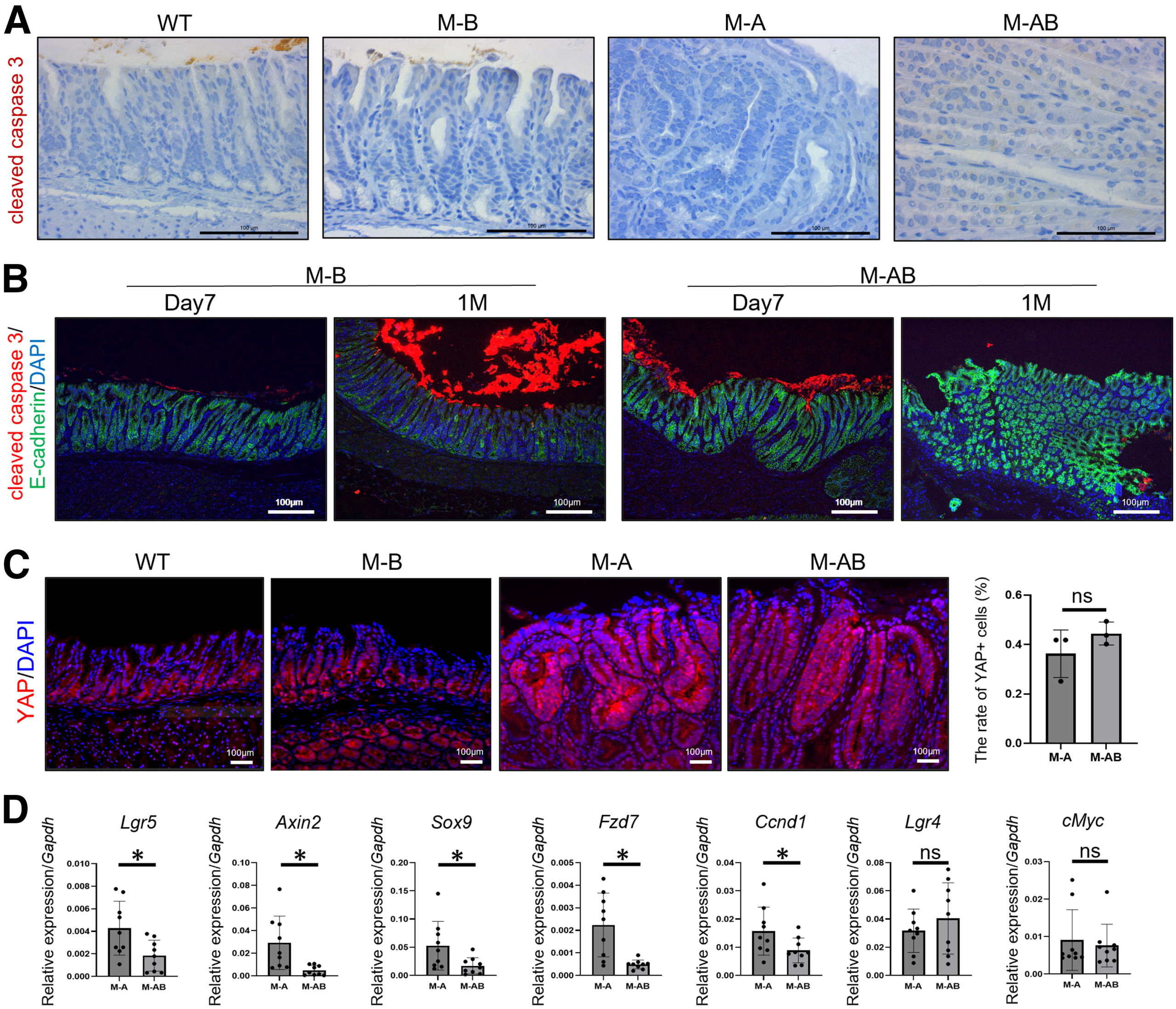

Figure 4. Bcl-xL deletion suppresses Wnt signaling in APC-deficient gastric epithelium. (*A*) Cleaved caspase 3 staining in WT, M-B, M-A, and M-AB mice (Representative images for n = 3 mice/group). (*B*) Cleaved caspase 3 (*red*) and E-cadherin (*green*) staining in M-B and M-AB mice 7 days and 1 month after TAM induction (representative images for n = 3 mice/group). (*C*) YAP staining in WT, M-B, M-A, and M-AB mice. The rates of YAP-positive cells were quantified (n = 3/group). The *P* value was calculated using a *t* test. (*D*) RT-PCR of *Lgr5, Axin2, Sox9, Fzd7, Ccnd1, Lgr4, and cMyc* in M-A and M-AB mice (n = 9 mice/group). The *P* value was calculated using a *t* test. Scale bars, 100 μm. Mean ± standard error of the mean. *$P < .05$. DAPI, 4′,6-diamidino-2-phenylindole.

organoid models (Figure 7*D*). Finally, we confirmed that there was no difference in apoptosis occurrence between KPA and KPAB xenografts (Figure 7*E*).

These results collectively demonstrate that Bcl-xL promotes gastric tumorigenesis by enhancing Wnt pathway activity and supporting tumor cell proliferation, both in vitro and in vivo.

Negative Regulation of Hepatocyte Nuclear Factor 4 α by B-Cell Lymphoma–Extra-Large Contributes to the Activation of Wnt–β-Catenin Signaling

To comprehensively assess gene expression changes associated with Bcl-xL deficiency, RNA sequencing was performed using RNA extracted from WT, KPA, and KPAB organoids. Principal component analysis (PCA) demonstrated distinct clustering of each group, indicating unique transcriptomic profiles (Figure 8*A*). A focused heatmap of key gene sets (Figure 8*B*) revealed higher expression of Wnt-related genes in KPA organoids compared with WT and KPAB organoids. Within the Bcl2 family, *Bcl2l1* was downregulated in KPAB organoids. Furthermore, the differentiation markers, including *Muc6* and *Tff2* for neck cell lineage and *Muc5ac* and *Tff1* for pit cell lineage, are upregulated in KPAB organoids, compared with KPA organoids (Figure 8*C*). Gene Set Enrichment Analysis (GSEA) revealed downregulation of gene sets associated with the WNT pathway and epithelial cell proliferation in KPAB organoids, consistent

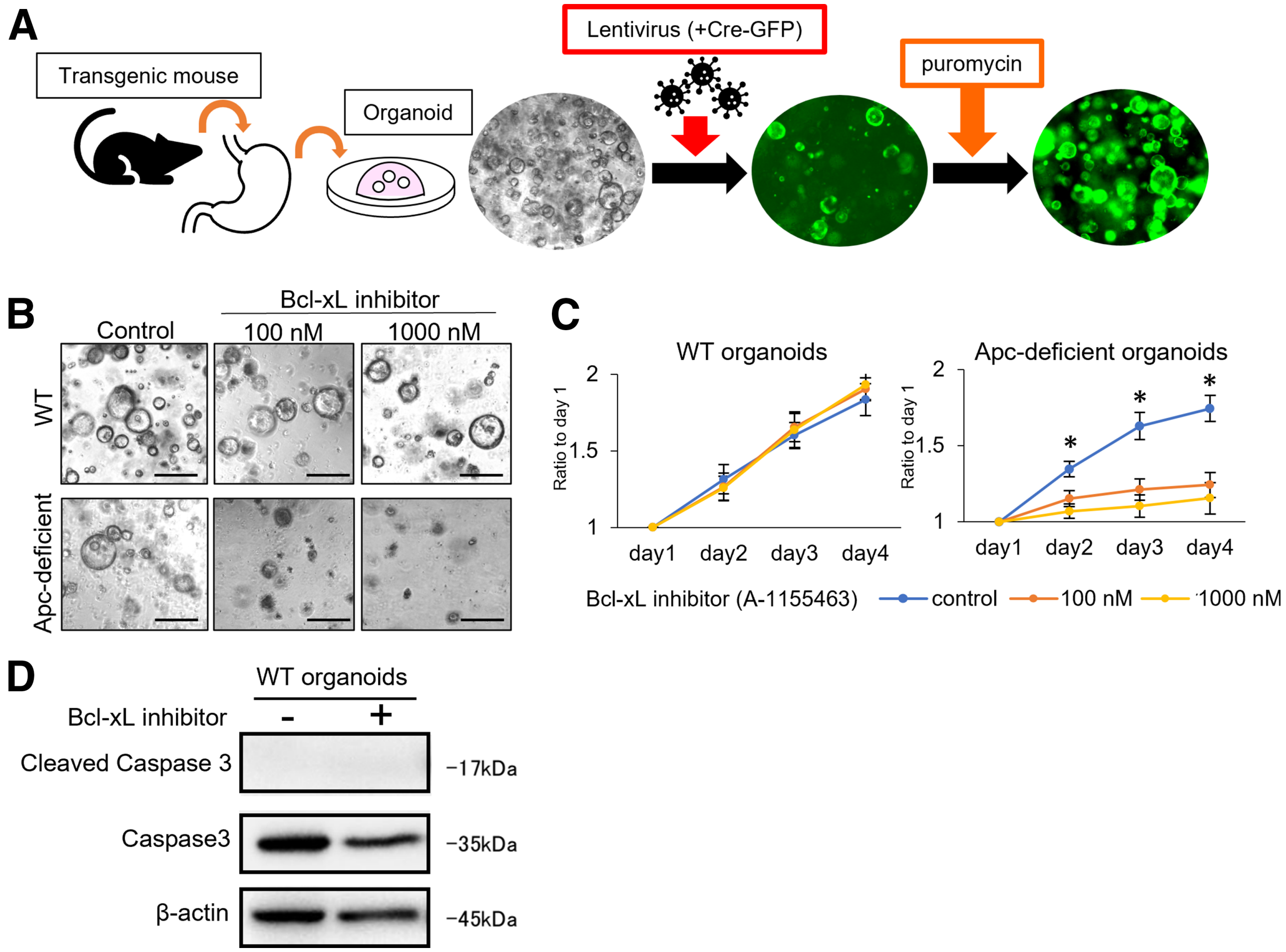

Figure 5. Apc-deficient gastric organoids are sensitive to Bcl-xL inhibition. (*A*) The schema of the lentivirus-infected organoid development. (*B–D*) Treatment with Bcl-xL inhibitor (A-1155463) in WT and Apc organoids. (*B*) The representative images of the organoids. (*C*) The transitions of organoid diameters were quantified (n = 10/group). The *P* value was calculated using a *t* test. (*D*) Western blotting with cleaved caspase 3, caspase 3, and β actin in WT organoids with and without Bcl-xL inhibitor. Scale bars, 100 μm. Mean ± standard error of the mean. *$P < .05$.

with the results of other experiments (Figure 8*D*). These findings suggest that loss of Bcl-xL impairs WNT-driven epithelial proliferation, which may contribute to the reduced growth capacity observed in KPAB organoids.

Comparing the top 100 differentially expressed genes across the 3 groups, *Hnf4a* exhibited decreased expression in KPA organoids and increased expression in KPAB organoids (Figure 8*E* and *F* and Supplementary Table 1). *Hnf4a* is a transcription factor reported to regulate the Wnt signaling and cell proliferation pathways.[50] To assess its functional role, we overexpressed *Hnf4a* in KPA organoids using adenoviral vectors (Figure 9*A*). *Hnf4a*-overexpression (OE) suppressed cell proliferation in KPA organoids (Figure 9*B*). To thoroughly characterize transcriptional differences, we performed RNA sequencing on control and *Hnf4a*-OE KPA organoids. PCA revealed distinct clustering of the 2 groups, indicating unique transcriptomic profiles (Figure 9*C*). Heatmap visualization further highlighted differentially expressed gene patterns (Figure 9*D*). GSEA demonstrated that *Hnf4a*-OE significantly inhibited the activation of WNT pathway, with the down-regulation of Wnt target genes (Figure 9*E* and *F*). RT-PCR analysis also revealed that *Hnf4a*-OE KPA organoids exhibited reduced expression of several Wnt target genes, including *Sox9, Axin2,* and *Fzd7*, compared with control KPA organoids (Figure 9*G*). These results suggest that Bcl-xL negatively regulates the *Hnf4a* expression, which activates the Wnt–β-catenin pathway and enhances the proliferation.

Negative Regulation of Peroxisome Proliferator–Activated Receptor γ Expression by B-Cell Lymphoma–Extra-Large Contributes to the Tumor Growth

Focusing again on the GSEA of RNA sequencing for KPA, and KPAB organoids, the HALLMARK CHOLESTEROL HOMEOSTASIS gene set was significantly enriched in KPAB organoids, and *Pparg* was notably upregulated in KPAB organoids (Figure 10*A* and Supplementary Table 3), along with the other metabolic pathways including bile acid, fatty acid, and xenobiotic ones. Both qPCR and western blotting validated the *Pparg* upregulation in KPAB organoids compared with KPA organoids (Figure 10*B* and *C* and

Supplementary Western blotting *F–G*). Additionally, immunohistochemical staining of xenograft sections revealed that GFP-positive cells from KPAB tumors strongly expressed PPARγ, whereas those from KPA tumors did not (Figure 10*D*). Tumor tissue in M-AB mice also showed increased expression of PPARγ compared with M-A mice. (Figure 10*E*).

Given the observed upregulation of *Pparg* in Bcl-xL–deficient contexts, we next examined whether forced expression of *Pparg* changes the gene expressions and

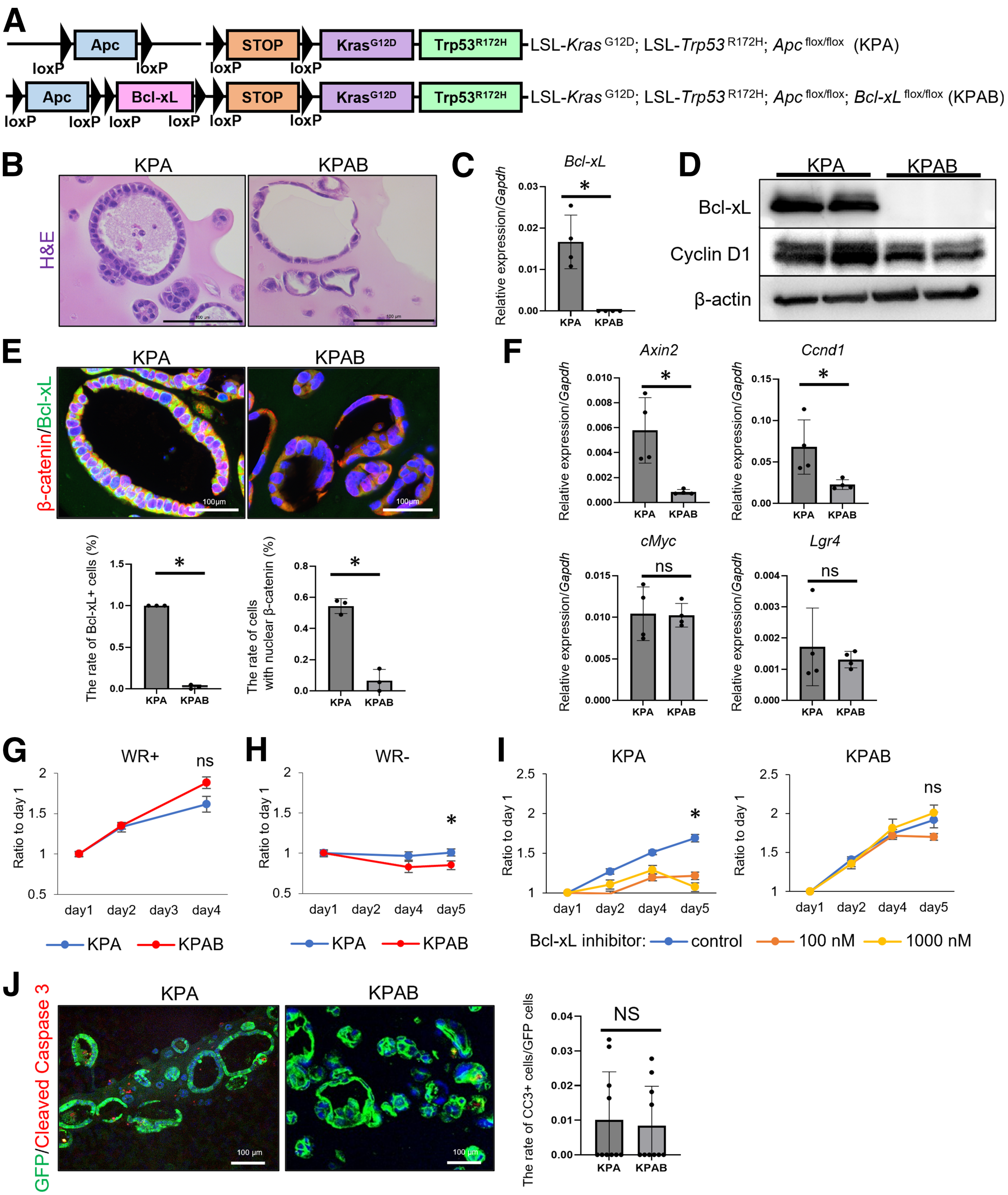

tumor growth. KPA organoids were infected with either *Pparg*-OE adenovirus or a control adenovirus. Fluorescent immunostaining and qPCR showed clear upregulation of *Pparg* expression in organoids infected with *Pparg*-OE adeno virus than control adeno virus (Figure 11*A* and *B*). Next, to comprehensively evaluate transcriptional changes, RNA sequencing was performed on KPA organoids and *Pparg*-OE KPA organoids. PCA revealed distinct clustering of the 2 groups, indicating unique transcriptomic profiles (Figure 11*C*). GSEA demonstrated that *Pparg*-OE significantly activated the metabolic pathways, such as fatty acid, bile acid, cholesterol, and xenobiotic ones, with the upregulation of lipid metabolism-related genes (Figure 11*D* and *E*). Measurement of organoid diameter over time revealed significant reduction of organoid growth in *Pparg*-OE KPA organoids compared with control KPA organoids (Figure 11*F*). GSEA revealed that *Pparg*-OE suppresses WNT pathway activation (Figure 11*G*), whereas *Hnf4a* expression is unaffected (Figure 11*H*), indicating that hepatocyte nuclear factor 4 α (HNF4A) does not act downstream of PPARγ and that PPARγ directly modulates WNT signaling. Moreover, *Hnf4a*-OE significantly impacted gene sets associated with similar metabolic pathways including fatty acid, bile acid, cholesterol, and xenobiotic ones (Figure 11*I* and Supplementary Table 4) and slightly upregulate the *Pparg* expression (Figure 11*J*). These data suggest that Bcl-xL may partially regulate PPARγ expression through HNF4A (Figure 11*K*).

The Relationship Among B-Cell Lymphoma–Extra-Large, Peroxisome Proliferator–Activated Receptor γ, and WNT Pathway Validated by Single-Cell RNA Sequencing of Mice With Gastric Cancer

To validate the relationship between Bcl-xL, PPARγ, and the WNT pathway in gastric cancer, we performed single-cell RNA sequencing analyses using both mouse GC datasets.

For mouse GC, we utilized antral tumor data from Gp130$^{Y757F/Y757F}$ mice (GSE217498).[51] After manual clustering, epithelial cell clusters (clusters 2, 6, 10, and 12) positive for Epcam were isolated and further subdivided into *Bcl2l1*-positive and *Bcl2l1*-negative populations (Figure 12*A*–*D*). *Bcl2l1* expression was used as a surrogate for Bcl-xL expression, as Bcl-xL is the predominant isoform encoded by *Bcl2l1* in mice.[52] *Bcl2l1*-positive cells exhibited reduced *Pparg* expression (Figure 12*E*), and GSEA using Hallmark gene sets revealed significant downregulation of fatty acid, bile acid, and cholesterol metabolism pathways (Figure 12*F*). In contrast, WNT-related gene sets were significantly enriched in the *Bcl2l1*-positive cell population (Figure 12*G*).

Collectively, these single-cell transcriptomic analyses demonstrate a conserved association between *Bcl2l1* expression, suppression of metabolic programs, and activation of WNT signaling in mouse GC, supporting the translational relevance of our Bcl-xL–deficient models.

B-Cell Lymphoma–Extra-Large and Peroxisome Proliferator–Activated Receptor γ Are Prognostic Factors in Human Gastric Cancer

To assess whether the findings from mouse models in vivo and in vitro are also relevant to human GC, we examined the expression of Bcl-xL (or *BCL2L1*) and *PPARG* in human GC samples.

First, immunofluorescence staining for Bcl-xL was performed using a human GC tissue array. A total of 72 cases were scored based on the intensity of Bcl-xL staining and stratified by clinical stage, showing that Bcl-xL expression increased with tumor stage, suggesting a correlation between Bcl-xL expression and cancer progression (Figure 13*A*).

Using the UALCAN application and The Cancer Genome Atlas (TCGA) database,[53] we next evaluated *BCL2L1* expression in normal gastric mucosa and GC and found that *BCL2L1* expression was elevated in GC compared with normal gastric mucosa (Figure 13*B*). *BCL2L1* expression was used as a surrogate for Bcl-xL expression, as Bcl-xL is the predominant isoform encoded by *BCL2L1* in human normal tissue and cancers.[54]

Next, we analyzed gene expression profiles in 450 GC samples from TCGA with the Phantasus application.[55] Cases were divided into *BCL2L1*-high and *BCL2L1*-low groups, and expression levels of Wnt target genes previously identified in mouse and organoid models (including *CCND1, LGR5, AXIN2,* and *SOX9*) were compared. This analysis revealed that *CCND1, LGR5, AXIN2*, and *SOX9* were significantly upregulated in the *BCL2L1*-high group, supporting the idea that Bcl-xL enhances Wnt activation in human GC (Figure 13*C*).

Figure 6. (***See previous page***). **Bcl-xL enhances the proliferation in the Wnt-dependent manner in organoid models.** (*A*) Gene construction schema showing KPA and KPAB organoid models. (*B*) Hematoxylin and eosin staining in KPA and KPAB organoids. (*C*) RT-PCR of *Bcl2l1* (Bcl-xL) in KPA and KPAB organoids (n = 4/group). The *P* value was calculated using a *t* test. (*D*) Western blotting of Bcl-xL and cyclin D1 in KPA and KPAB organoids are shown (n = 2/group). (*E*) Bcl-xL (*green*) andβ-catenin (*red*) staining in KPA and KPAB organoids. The rates of Bcl-xL+ cells and β-catenin–nuclear-translocated cells were quantified (n = 3 organoids/group). The *P* value was calculated using a *t* test. (*F*) RT-PCR of *Axin2, Ccnd1, cMyc*, and *Lgr4* in KPA and KPAB organoids (n = 4/group). The *P* value was calculated using a *t* test. (*G*) The culture of KPA and KPAB organoids in standard medium. The ratios of organoid diameters relative to those on day 1 were quantified (n = 10/group). The *P* value was calculated using a *t* test. (*H*) The culture of KPA and KPAB organoids in medium lacking Wnt3a, R-spondin 3, and growth factors. The ratios of organoid diameters relative to those on day 1 were quantified (n = 10 per group). The *P* value was calculated using a *t* test. (*I*) Treatment with Bcl-xL inhibitor (100 nM and 1000 nM) in KPA and KPAB organoids. The ratios of organoid diameters relative to those on day 1 were quantified (n = 10 per group). The *P* value was calculated using a *t* test. (*J*) GFP (*green*) and cleaved caspase 3 (*red*) staining of KPA and KPAB organoids in the growth factor-free condition. The rates of cleaved caspase 3 cells were quantified (n = 10 organoids/group). The *P* value was calculated using a *t* test. Scale bars, 100 μm. Mean ± standard error of the mean. *$P < .05$.

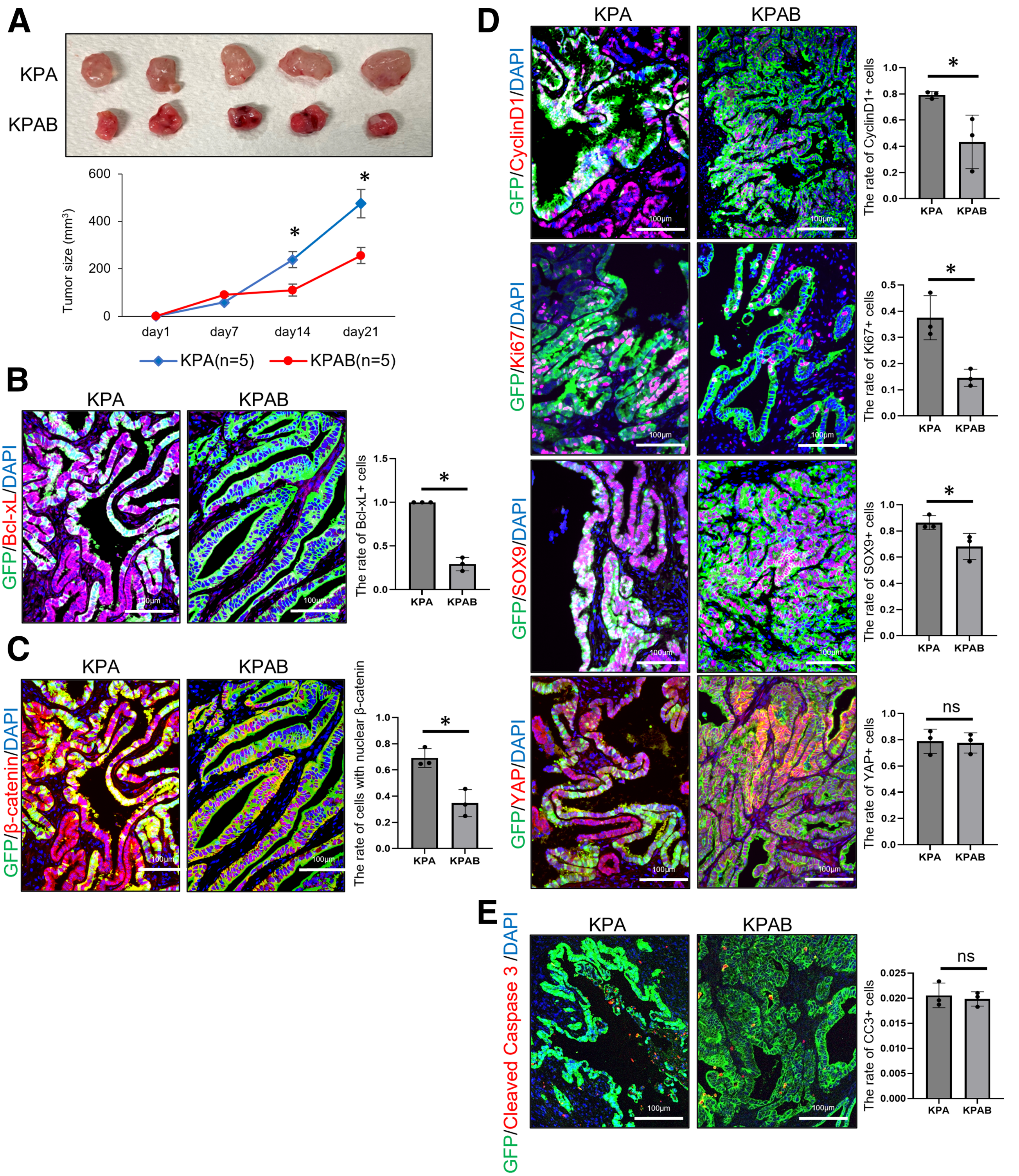

Figure 7. Bcl-xL deletion suppresses the proliferation via inactivation of Wnt/β-catenin pathway in xenograft models. (*A*) Gross findings of the KPA and KPAB xenografts. The tumor sizes were quantified (n = 5/group). The *P* value was calculated using a *t* test. (*B*) GFP (*green*) and Bcl-xL (*red*) staining in KPA and KPAB xenografts. The rates of Bcl-xL + cells were quantified (n = 3/group). The *P* value was calculated using a *t* test. (*C*) GFP (*green*) andβ-catenin (*red*) staining in KPA and KPAB organoids. The rates of β-catenin–nuclear-translocated cells were quantified (n = 3/group). The *P* value was calculated using a *t* test. (*D*) GFP (*green*) and cyclin D1 (*red*), GFP (*green*) and SOX9 (*red*), GFP (*green*) and Ki67 (*red*), and GFP (*green*) and YAP (*red*) staining in KPA and KPAB xenograft. The rates of cyclin D1-, SOX9-, Ki67-, and YAP-positive cells were quantified (n = 3/group). The *P* value was calculated using a *t* test. (*E*) GFP (*green*) and cleaved caspase 3 (*red*) staining in KPA and KPAB xenografts. The rates of cleaved caspase 3–positive cells were quantified (n = 3/group). The *P* value was calculated using a *t* test. Scale bars, 100 μm. Mean ± standard error of the mean. *$P < .05$. DAPI, 4′,6-diamidino-2-phenylindole.

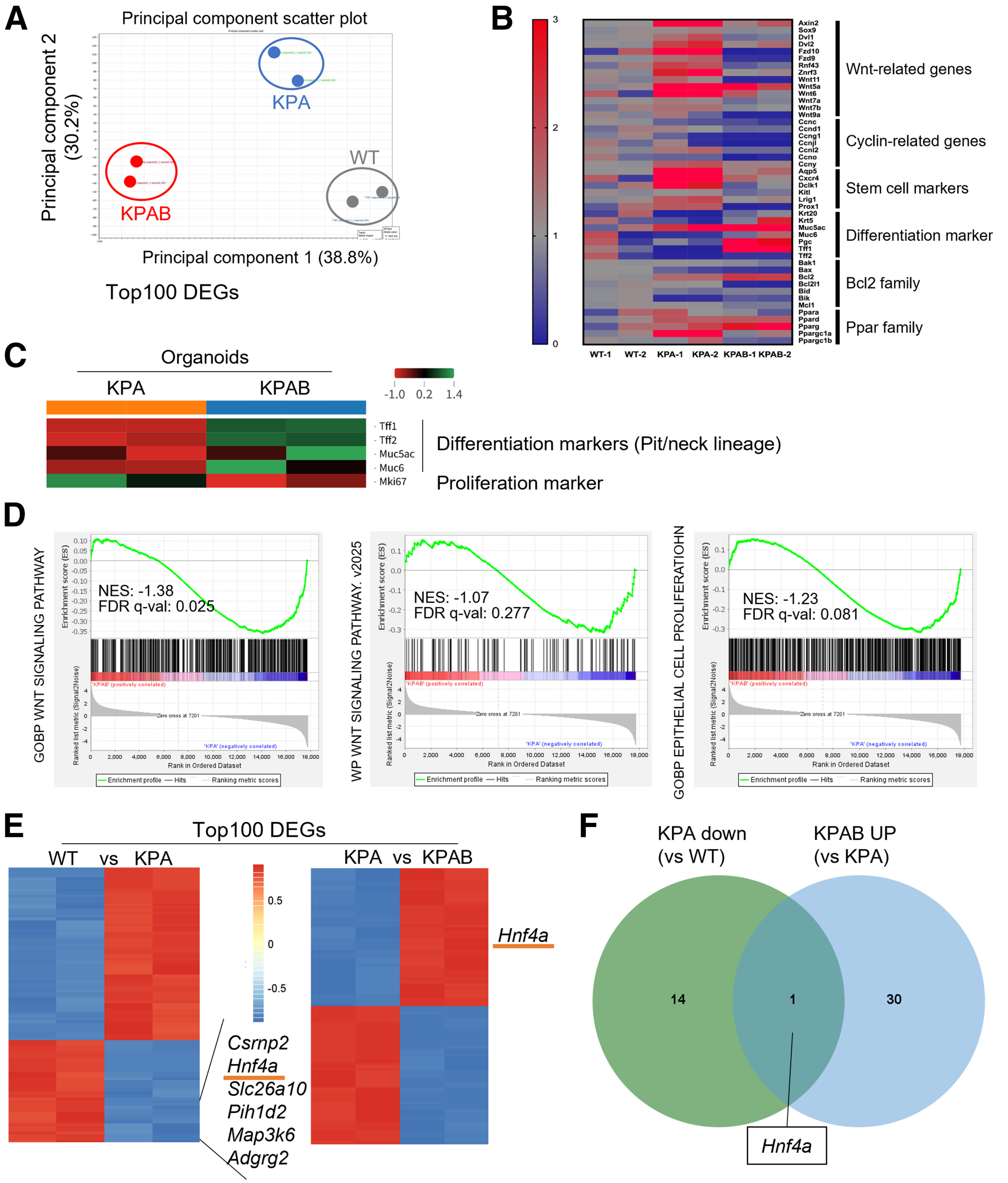

Figure 8. Comparative bulk RNA sequencing analysis of WT, KPA, and KPAB gastric organoids. (*A–F*) Bulk RNA sequencing comparing WT, KPA, and KPAB organoids (n = 2/group). (*A*) PCA plots. (*B*) Heatmap of gene expression. (*C*) Heatmap showing the *Tff1, Tff2, Muc5ac, Muc6,* and *Mki67* expression. (*D*) Sigmoid curve showing the result of the GSEA with Gene Ontology Biological Process (GOBP) WNT SIGNALING PATHWAY, WP WNT SIGNALING PATHWAY. v2025, and GOBP EPITHELIAL CELL PROLIFERATIOHN gene sets. (*E*) Heatmaps showing the top 100 differentially expressed genes (DEGs) across the 3 groups. (*F*) Venn diagram of the downregulated DEGs in KPA organoids and upregulated DEGs in KPAB organoids.

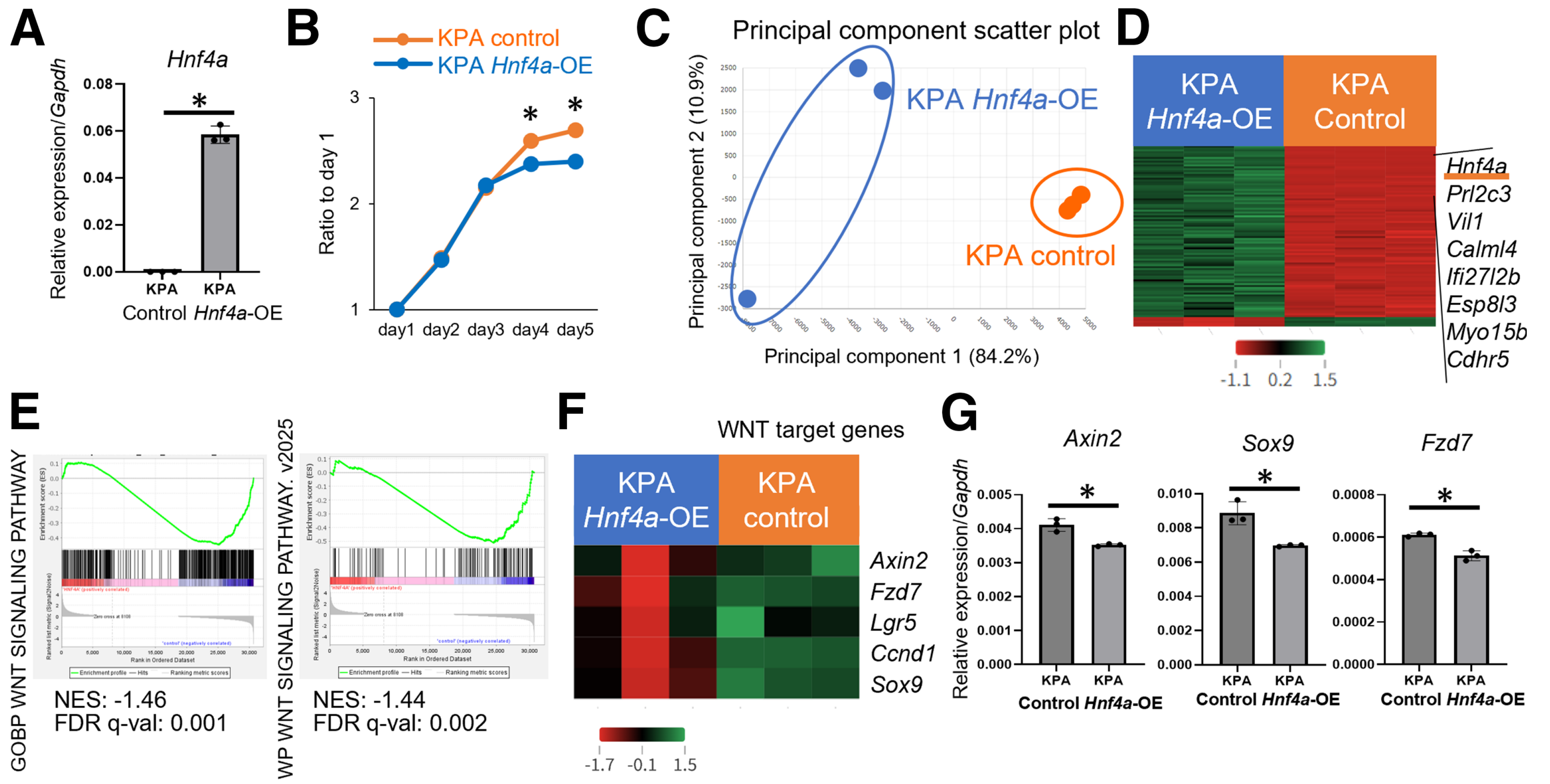

Figure 9. Bcl-xL–mediated suppression of *Hnf4a* expression promotes activation of the Wnt–β-catenin pathway. (*A*) RT-PCR of *Hnf4a* in KPA organoids and KPA organoids overexpressing *Hnf4a* (n = 3/group). The *P* value was calculated using a *t* test. (*B*) The infection of KPA organoids with *Hnf4a*-overexpressing adenovirus. The ratios of organoid diameters relative to those on day 1 were quantified (n = 10 per group). The *P* value was calculated using a *t* test. (*C–F*) Bulk RNA sequencing comparing KPA organoids and KPA organoids overexpressing *Hnf4a* (n = 3/group). (*C*) PCA plot, (*D*) Heat map of top 100 differentially expressed genes (DEGs). (*E*) GSEA with Gene Ontology Biological Process (GOBP) WNT SIGNALING PATHWAY, WP WNT SIGNALING PATHWAY. v2025, and GOBP EPITHELIAL CELL PROLIFERATIOHN gene sets. (*F*) Heatmap of the WNT-target genes. (*G*) RT-PCR of *Sox9*, *Axin2*, and *Fzd7* in KPA organoids and KPA organoids overexpressing *Hnf4a* (n = 3/group). The *P* value was calculated using a *t* test. Mean ± standard error of the mean. *$P < .05$.

Next, we analyzed the gene expression profiles in 412 GC samples from the PanCancer Atlas and analyzed the association between *BCL2L1* and several gene sets. GSEA showed the downregulation of metabolic pathways including bile acid and fatty acid metabolism, compatible with our findings from mice experiments (Figure 13*D* and Supplementary Table 5).

We then explored the prognostic significance of *BCL2L1* and *PPARG* expression using the Kaplan–Meier Plotter, a database linking gene expression to patient survival outcomes. In a cohort of 875 patients with GC, higher *BCL2L1* expression was associated with a poorer prognosis, whereas higher *PPARG* expression correlated with better prognosis (Figure 13*E*). Furthermore, Spearman correlation analysis revealed a negative correlation between *BCL2L1* and *PPARG* expression ($r = -0.1591$; $P < .0001$), suggesting a potential regulatory relationship between the 2 genes (Figure 13*F*).

For human GC, we utilized the Single-Cell Atlas of human GC (GSE239676).[56] GCs with high *BCL2L1* expression showed significant enrichment of WNT pathway–related gene sets and of genes downregulated in *Hnf4a*-OE KPA organoids, whereas gene sets related to bile acid metabolism were downregulated, consistent with the findings in the mouse model.

To examine whether our findings may also apply to other gastrointestinal malignancies, we performed similar analyses using the TCGA database for esophageal cancer and colorectal cancer. In esophageal cancer, *BCL2L1* expression was particularly elevated in esophageal adenocarcinoma compared with normal mucosa and squamous cell carcinoma (Figure 14*A*). Esophageal adenocarcinomas with high *BCL2L1* expression showed activation of the WNT signaling pathway and reduced lipid metabolism compared with those with low *BCL2L1* expression (Figure 14*B*). In colorectal cancer, *BCL2L1* expression was also increased compared with normal mucosa (Figure 14*C*), and patients with *BCL2L1*-high and *PPARG*-low tumors had a worse prognosis than those with *BCL2L1*-low and *PPARG*-high tumors, respectively (Figure 14*D*). GSEA further demonstrated that *BCL2L1*-high colorectal cancers exhibited activation of the WNT signaling pathway and suppression of fatty acid metabolism compared with *BCL2L1*-low tumors (Figure 14*E*). Taken together, these results indicate that the association between *BCL2L1* expression, WNT signaling, and metabolic pathways observed in GC may also be present in esophageal adenocarcinoma and colorectal cancer.

Finally, we investigated the potential clinical relevance of PPARγ activation in GC prevention by analyzing data from 29,905 hospitalized diabetic patients in the Diagnosis Procedure Combination database. Since pioglitazone, a PPARγ agonist, is commonly used to treat diabetes, a known risk factor for GC, this analysis was restricted to

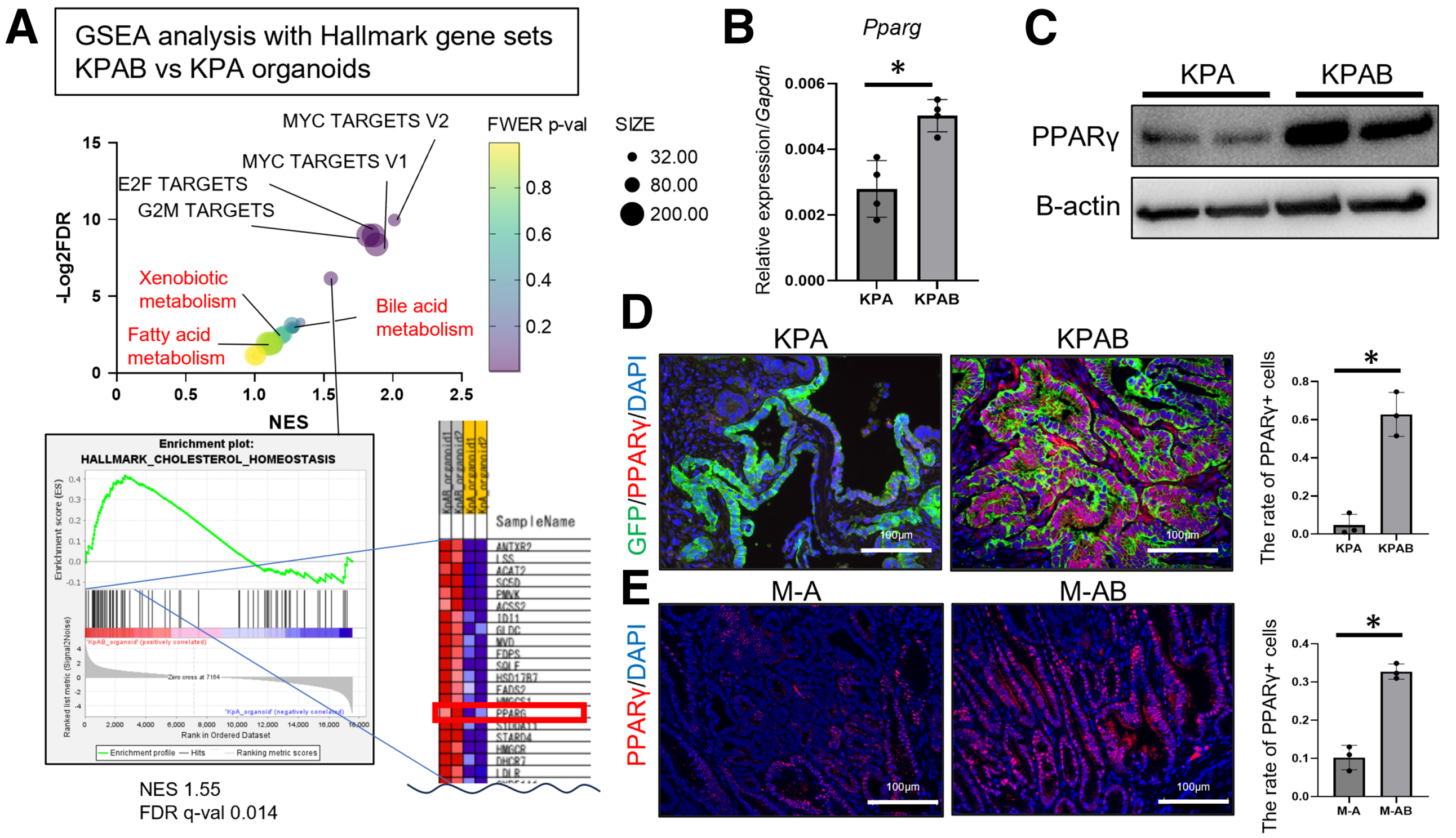

Figure 10. Bcl-xL suppresses PPARγ expression and multiple gene sets involved in metabolic pathways. (*A*) Bubble plots showing the GSEA analysis with Hallmark gene sets. Sigmoid curve showing the result with CHOLESTEROL HOMEOSTASIS gene set. (*B*) RT-PCR of *Pparg* in KPA and KPAB organoids (n = 4/group). The *P* value was calculated using a *t* test. (*C*) Western blotting of PPARγ in KPA and KPAB organoids (n = 2/group). (*D*) GFP (*green*) and PPARγ (*red*) staining in KPA and KPAB xenografts. The rates of PPARγ-positive cells were quantified (n = 3/group). The *P* value was calculated using a *t* test. (*E*) PPARγ (*red*) staining in M-A and M-AB mice. The rates of PPARγ-positive cells were quantified (n = 3/group). The *P* value was calculated using a *t* test. Scale bars, 100 μm. Mean ± standard error of the mean. **P* < .05. DAPI, 4′,6-diamidino-2-phenylindole.

diabetic patients (Table 1). Multivariate analysis adjusted for confounders showed that oral pioglitazone use was associated with a significantly reduced risk of GC (adjusted odds ratio [OR], 0.74; 95% confidence interval, 0.56–0.99; $P = .0419$).

Collectively, these findings suggest that Bcl-xL expression increases with tumor progression and promotes tumorigenesis via Wnt pathway activation in human gastric cancer, while PPARγ acts as a tumor suppressor negatively regulated by Bcl-xL. Moreover, activation of PPARγ by pioglitazone may provide a protective effect against GC development in diabetic patients.

Discussion

In this study, we demonstrate that Bcl-xL plays a previously unrecognized role in promoting gastric tumorigenesis beyond its canonical antiapoptotic function. Using genetically engineered mouse models, tumor organoids, and human clinical datasets, we show that Bcl-xL enhances GC progression primarily by augmenting Wnt–β-catenin signaling and tumor cell proliferation. Mechanistically, Bcl-xL negatively regulates the transcription factors HNF4A and PPARγ, thereby linking stem cell signaling, transcriptional control, and metabolic regulation during gastric tumor development.

Traditionally, Bcl-xL has been characterized as a key antiapoptotic member of the BCL2 family, contributing to tumor cell survival under stress conditions.[57] However, accumulating evidence suggests that BCL2 family proteins also exert noncanonical functions, including regulation of mitochondrial metabolism, transcriptional programs, and cell–cycle progression.[57] Our findings substantially expand this paradigm by identifying Bcl-xL as a positive regulator of oncogenic Wnt signaling in gastric epithelium. Notably, Bcl-xL deletion did not induce apoptosis in either normal or neoplastic gastric tissues, indicating that its tumor-promoting effects in this context are largely independent of cell death suppression. Instead, loss of Bcl-xL consistently attenuated proliferation, Wnt target gene expression, and tumor growth in vivo and in vitro, highlighting a novel and apoptosis-independent oncogenic function of Bcl-xL.

Our transcriptomic analyses revealed that Bcl-xL orchestrates broader transcriptional and metabolic programs through negative regulation of HNF4A and PPARγ. HNF4A is a key epithelial differentiation factor that has been reported to antagonize Wnt signaling and restrict proliferation in gastrointestinal tissues.[50] Restoration of HNF4A expression in

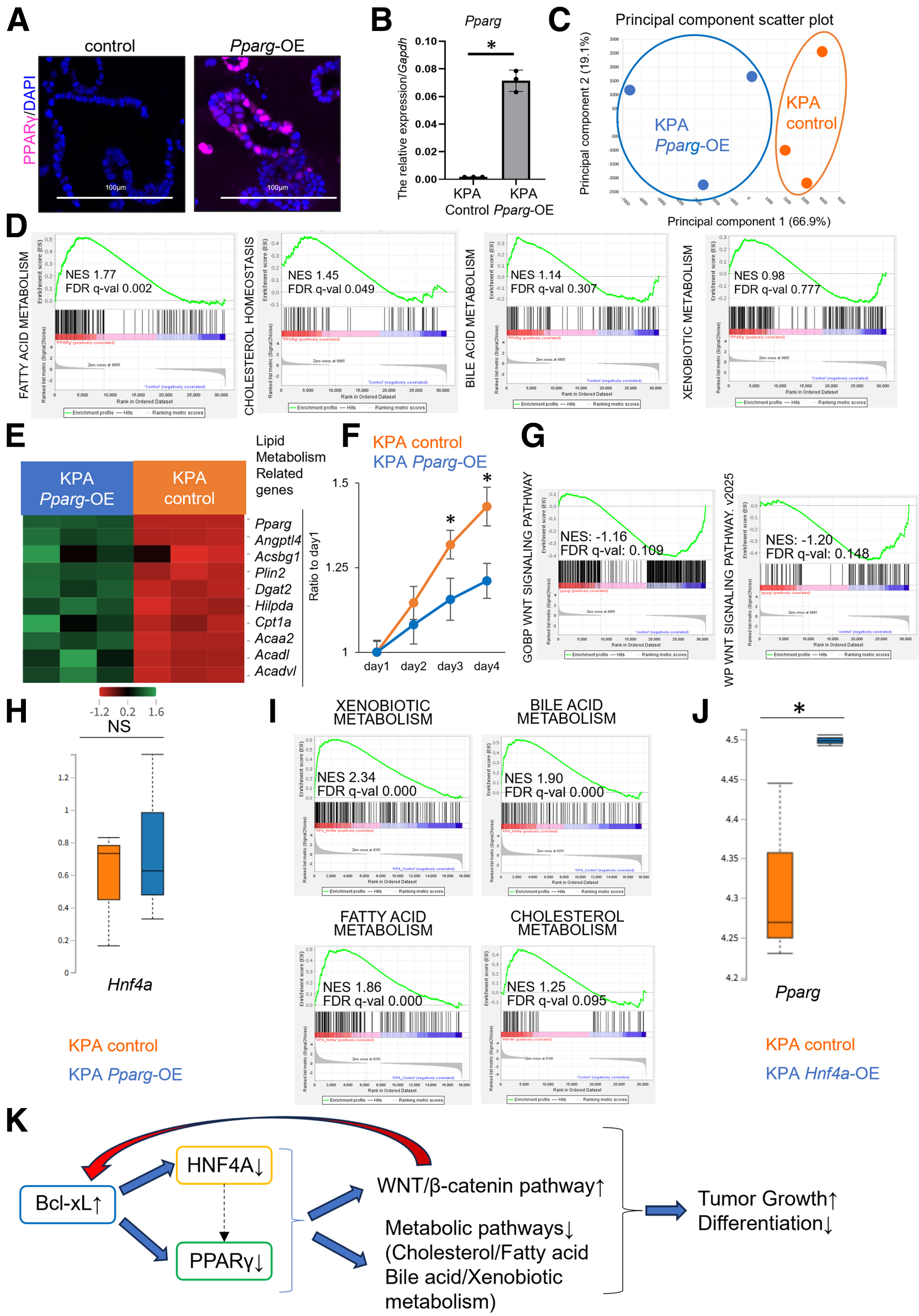
A
control
Pparg-OE
PPARγ/DAPI
100μm
B
Pparg
The relative expression/Gapdh
KPA Control
KPA Pparg-OE
C
Principal component scatter plot
Principal component 2 (19.1%)
Principal component 1 (66.9%)
KPA Pparg-OE
KPA control
D
FATTY ACID METABOLISM
NES 1.77
FDR q-val 0.002
CHOLESTEROL HOMEOSTASIS
NES 1.45
FDR q-val 0.049
BILE ACID METABOLISM
NES 1.14
FDR q-val 0.307
XENOBIOTIC METABOLISM
NES 0.98
FDR q-val 0.777
E
KPA Pparg-OE
KPA control
Lipid Metabolism Related genes
Pparg
Angptl4
Acsbg1
Plin2
Dgat2
Hilpda
Cpt1a
Acaa2
Acadl
Acadvl
-1.2 0.2 1.6
F
KPA control
KPA Pparg-OE
Ratio to day1
day1 day2 day3 day4
G
GOBP WNT SIGNALING PATHWAY
NES: -1.16
FDR q-val: 0.109
WP WNT SIGNALING PATHWAY. v2025
NES: -1.20
FDR q-val: 0.148
H
NS
Hnf4a
KPA control
KPA Pparg-OE
I
XENOBIOTIC METABOLISM
NES 2.34
FDR q-val 0.000
BILE ACID METABOLISM
NES 1.90
FDR q-val 0.000
FATTY ACID METABOLISM
NES 1.86
FDR q-val 0.000
CHOLESTEROL METABOLISM
NES 1.25
FDR q-val 0.095
J
Pparg
KPA control
KPA Hnf4a-OE
K
Bcl-xL↑
HNF4A↓
PPARγ↓
WNT/β-catenin pathway↑
Metabolic pathways↓
(Cholesterol/Fatty acid
Bile acid/Xenobiotic
metabolism)
Tumor Growth↑
Differentiation↓

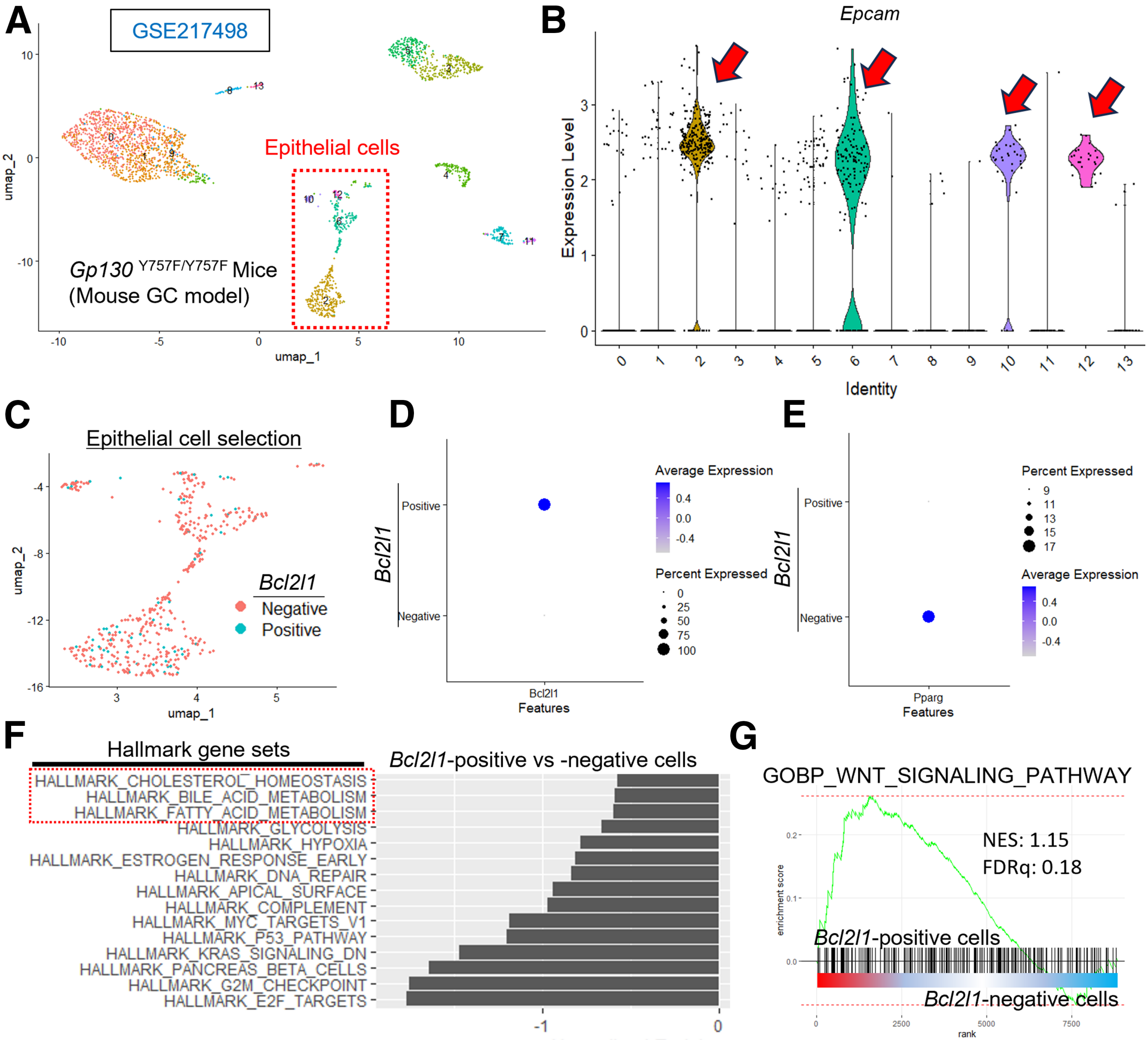

Figure 12. Analysis of Bcl-xL and PPARγ expression and WNT pathway activity in single-cell RNA sequencing of mouse GC. (*A–G*) Single-cell RNA sequencing analysis of mouse GC (Gp130$^{Y757F/Y757F}$ mice). (*A*) Uniform Manifold Approximation and Projection (UMAP) visualization of single-cell transcriptomic profiles. (*B*) Violin plot showing *Epcam* expression across clusters. *Red arrows* show the *Epcam*-positive clusters. (*C*) UMAP highlighting *Bcl2l1*-positive and -negative cells within epithelial cell clusters. (*D*) Dot plot showing *Bcl2l1* expression. (*E*) Dot plot showing *Pparg* expression. (*F*) GSEA using Hallmark gene sets comparing *Bcl2l1*-positive and -negative cells. (*G*) Sigmoid curve showing GSEA results for the Gene Ontology Biological Process (GOBP) WNT signaling pathway.

Figure 11. (***See previous page***). **Bcl-xL–mediated suppression of PPARγ expression promotes tumor growth.** (*A–H*) The infection of KPA organoids with *Pparg*-overexpressing adenovirus. (*A*) PPARγ staining in organoids infected with control adeno virus and *Pparg*-overexpressing adeno virus. (*B*) RT-PCR of *Pparg* expression (n = 3/group). The *P* value was calculated using a *t* test. (*C–H*) Bulk RNA sequencing comparing KPA organoids and KPA organoids overexpressing *Pparg* (n = 3/group). (*C*) PCA plot. (*D*) Sigmoid curve showing the result of the GSEA with gene sets for xenobiotic, bile acid, fatty acid, and cholesterol metabolism. (*E*) Heatmap of the lipid metabolism–related genes. (*F*) The ratios of organoid diameters relative to those on day 1 were quantified (n = 10 per group). The *P* value was calculated using a *t* test. (*G*) GSEA with Gene Ontology Biological Process (GOBP) WNT SIGNALING PATHWAY, WP WNT SIGNALING PATHWAY. v2025, and GOBP EPITHELIAL CELL PROLIFERATIOHN gene sets. (*H*) Boxplot of the *Hnf4a* expression based on the Bulk RNA sequencing comparing the control and *Pparg*-OE KPA organoids. The *P* value was calculated using a *t* test. (*I*) Sigmoid curve showing the result of the GSEA with gene sets for xenobiotic, bile acid, fatty acid, and cholesterol metabolism. (*J*) Boxplot of the *Pparg* expression based on the bulk RNA sequencing comparing the control and *Hnf4a*-OE KPA organoids. The *P* value was calculated using a *t* test. (*K*) Tumorigenic mechanism mediated by Bcl-xL. Scale bars, 100 μm. Mean ± standard error of the mean. *$P < .05$.

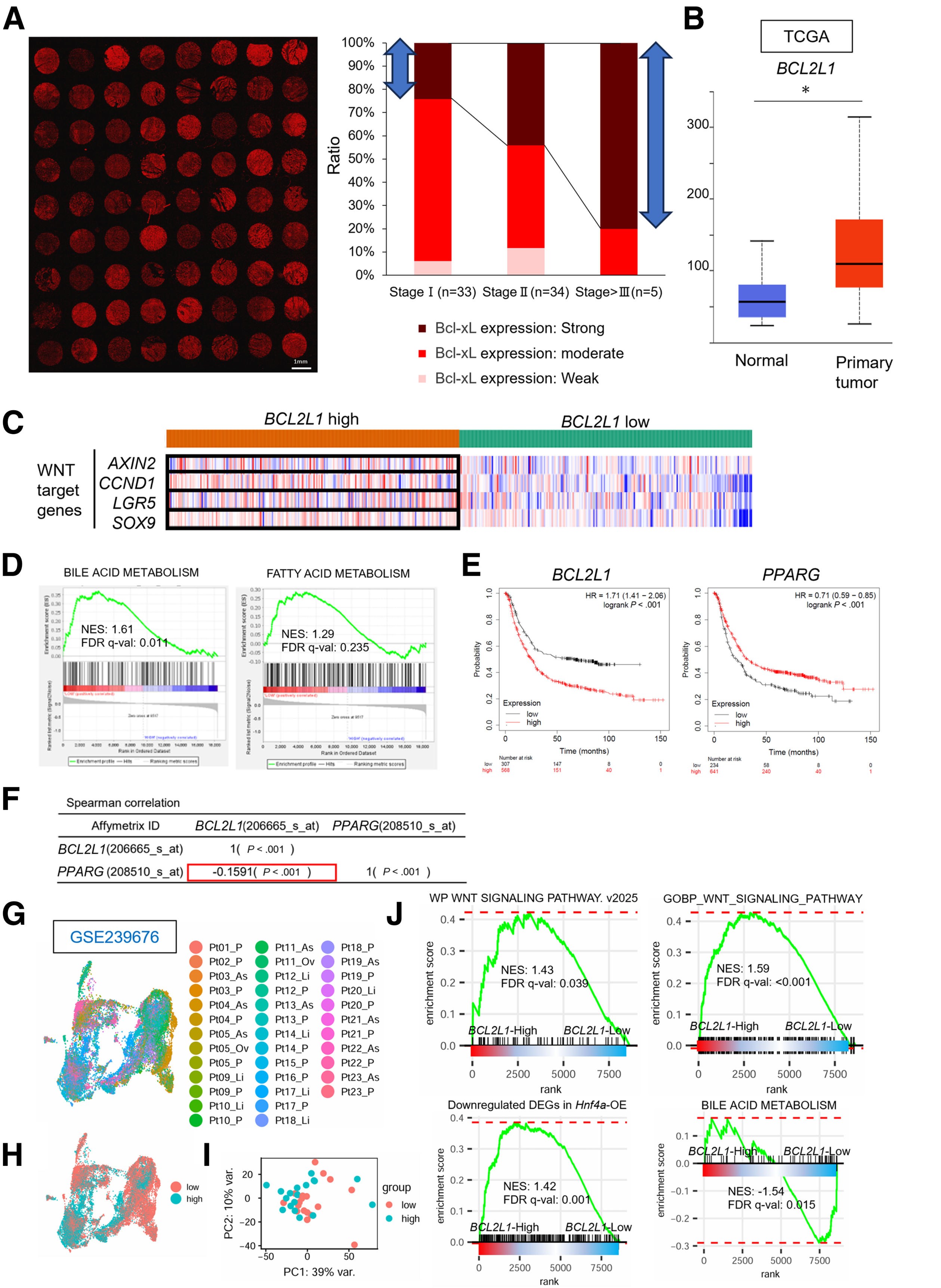
A
100%
90%
80%
70%
60%
50%
40%
30%
20%
10%
0%
Ratio
Stage Ⅰ (n=33) Stage Ⅱ (n=34) Stage>Ⅲ (n=5)
Bcl-xL expression: Strong
Bcl-xL expression: moderate
Bcl-xL expression: Weak
1mm
B
TCGA
BCL2L1
*
300
200
100
Normal
Primary tumor
C
BCL2L1 high
BCL2L1 low
WNT target genes
AXIN2
CCND1
LGR5
SOX9
D
BILE ACID METABOLISM
NES: 1.61
FDR q-val: 0.011
FATTY ACID METABOLISM
NES: 1.29
FDR q-val: 0.235
E
BCL2L1
HR = 1.71 (1.41 – 2.06)
logrank P < .001
PPARG
HR = 0.71 (0.59 – 0.85)
logrank P < .001
Probability
Expression
low
high
Time (months)
F
Spearman correlation
Affymetrix ID | BCL2L1(206665_s_at) | PPARG(208510_s_at)
BCL2L1(206665_s_at) | 1(P < .001) |
PPARG (208510_s_at) | -0.1591(P < .001) | 1(P < .001)
G
GSE239676
Pt01_P Pt02_P Pt03_As Pt03_P Pt04_As Pt04_P Pt05_As Pt05_Ov Pt05_P Pt09_Li Pt09_P Pt10_Li Pt10_P
Pt11_As Pt11_Ov Pt12_Li Pt12_P Pt13_As Pt13_P Pt14_Li Pt14_P Pt15_P Pt16_P Pt17_Li Pt17_P Pt18_Li
Pt18_P Pt19_As Pt19_P Pt20_Li Pt20_P Pt21_As Pt21_P Pt22_As Pt22_P Pt23_As Pt23_P
H
low
high
I
PC2: 10% var.
PC1: 39% var.
group
low
high
J
WP WNT SIGNALING PATHWAY. v2025
NES: 1.43
FDR q-val: 0.039
GOBP_WNT_SIGNALING_PATHWAY
NES: 1.59
FDR q-val: <0.001
Downregulated DEGs in Hnf4a-OE
NES: 1.42
FDR q-val: 0.001
BILE ACID METABOLISM
NES: -1.54
FDR q-val: 0.015
BCL2L1-High
BCL2L1-Low
enrichment score
rank

tumor organoids suppressed Wnt pathway activation and inhibited growth, indicating that Bcl-xL–mediated repression of HNF4A represents a critical mechanism by which Wnt signaling is sustained in gastric tumors. In parallel, PPARγ, an essential regulator of lipid metabolism and cellular differentiation,[58] was markedly upregulated upon Bcl-xL loss. PPARγ activation might directly suppress Wnt signaling and significantly inhibited tumor growth and altered metabolic gene expression, suggesting that Bcl-xL promotes tumor progression in part by constraining differentiation-associated metabolic pathways. The partial regulation of PPARγ by HNF4A further suggests a hierarchical network linking transcriptional control and metabolic state in GC cells.

Emerging evidence indicates that stem cell signaling, Wnt–β-catenin activity, and cellular metabolism are tightly interconnected during gastrointestinal tumorigenesis.[59] Wnt signaling is not only a central driver of epithelial stemness and proliferation but also a key regulator of metabolic reprogramming, promoting anabolic pathways that support rapid cell division. In gastric and intestinal epithelia, sustained Wnt activation has been shown to enhance glycolysis and lipid biosynthesis while suppressing differentiation-associated metabolic programs.[60,61] Conversely, transcription factors such as HNF4A and PPARγ are known to promote epithelial differentiation and metabolic homeostasis and have been reported to antagonize excessive Wnt signaling, either directly or indirectly, by regulating Wnt-related gene expression and lipid metabolic pathways.[50,58] Disruption of this balance through persistent Wnt activation coupled with repression of metabolic differentiation programs favors the maintenance of a highly proliferative, stem-like tumor cell state. In this context, our findings suggest that Bcl-xL functions as a molecular integrator that reinforces Wnt-driven proliferative signaling while concomitantly suppressing HNF4A- and PPARγ-mediated metabolic programs, including fatty lipid, bile acid, xenobiotic, and cholesterol metabolism. The attenuation of these differentiation-associated metabolic pathways compromises normal epithelial differentiation, thereby creating a permissive environment for gastric tumor growth.

Several previous studies have reported the expression patterns of Bcl-xL in gastrointestinal tissues. Regarding the stomach,[38,62] Bcl-xL is broadly expressed in nearly all epithelial cells in mice based on immunohistochemistry. In humans, Bcl-xL expression was increased in gastric tumor tissues compared with adjacent normal mucosa, while some degree of Bcl-xL expression was also observed in normal tissue. In other organs, immunohistochemical analysis demonstrated diffused Bcl-xL expression in both normal and tumor tissues of the human and mice colon.[42,63] In contrast, Bcl-xL expression was relatively low in WT pancreatic tissue but progressively increased during neoplastic progression, with stronger expression in pancreatic intraepithelial neoplasia 2–3 lesions and further elevation in pancreatic ductal adenocarcinoma for both human and mice.[40,64] Taken together, these previous reports suggest that, consistent with our findings, broad Bcl-xL expression is observed in normal gastrointestinal tissues, whereas Bcl-xL expression patterns can vary depending on the tissue context and disease stage.

In addition to the tumor-intrinsic context examined in this study, the role of Bcl-xL may vary depending on the inflammatory microenvironment. Under conditions of chronic gastric inflammation, such as *H pylori*-associated gastritis and autoimmune gastritis,[65,66] epithelial cells are exposed to increased oxidative stress, cytokine signaling, and immune-mediated injury. In such settings, antiapoptotic factors including Bcl-xL may play a more prominent role in maintaining epithelial survival and tissue integrity. Chronic inflammatory signaling pathways, including NF-κB activation and cytokine-mediated stress responses, may increase the reliance of epithelial cells on Bcl-xL–mediated survival mechanisms. Therefore, although our current model does not demonstrate a major role for Bcl-xL in regulating apoptosis in established tumors under noninflammatory conditions, its functional contribution may be context dependent and could be enhanced during inflammation-driven gastric tumorigenesis.

Although APC deletion results in constitutive activation of Wnt–β-catenin signaling, accumulating evidence indicates that Wnt signaling output is not binary but exists along a graded spectrum.[67] Even in APC-deficient contexts, transcriptional activity of β-catenin–dependent target genes can be further modulated by additional regulatory factors. In our system, Bcl-xL enhances Wnt–β-catenin signaling; however, we did not observe impaired organoid growth or morphologic features consistent with excessive Wnt activation. This suggests that Bcl-xL-mediated augmentation likely operates within a permissive signaling range rather than exceeding the threshold that

Figure 13. (***See previous page***). **Analysis of Bcl-xL/BCL2L1 and PPARγ expression in human gastric cancer database.** (*A*) Bcl-xL staining in human GC tissue array. The grade of the Bcl-xL positivity is quantified across the tumor stage (n = 72). (*B*) Box plot showing the *BCL2L1* expression comparing the normal stomach (n = 34) and primary tumor tissue (n = 415) based on the TCGA database. The *P* value was calculated using a *t* test. (*C*) The heat map of the *AXIN2*, *CCND1*, *LGR5*, and SOX9 expression comparing between high- and low- *BCL2L1* expressed GC based on the TCGA database (n = 225 vs 225). (*D*) Sigmoid curve showing the result of the GSEA with gene sets for bile acid and fatty acid metabolism, comparing between high- and low- *BCL2L1* expressed GC based on the TCGA database (n = 206 vs 206). (*E*) Survival analysis of patients with gastric cancer (n = 875) stratified by high vs low *BCL2L1* and high vs low *PPARG* expression. The *P* value was calculated using a log-rank test. (*F*) Analysis of Spearman's rank coefficient between *BCL2L1* and *PPARG* expression (n = 450). (*G–J*) The analysis of public single-cell RNA sequencing data for GC (GSE239676) comparing tumor epithelial samples with high *BCL2L1* expression with those with low *BCL2L1* expression (n = 37). (*G*) Uniform Manifold Approximation and Projection (UMAP) plot colored by GC patient IDs. (*H*) UMAP plot stratified on the basis of *BCL2L1* expression. (*I*) PCA plot. (*J*) GSEA with Gene Ontology Biological Process (GOBP) WNT SIGNALING PATHWAY, WP WNT SIGNALING PATHWAY. v2025, GOBP EPITHELIAL CELL PROLIFERATIOHN, and Hallmark bile acid metabolism gene sets and downregulated differentially expressed genes (DEGs) in *Hnf4a*-OE KPA organoid mice. Scale bars, 100 μm. Mean ± standard error of the mean. *$P < .05$.

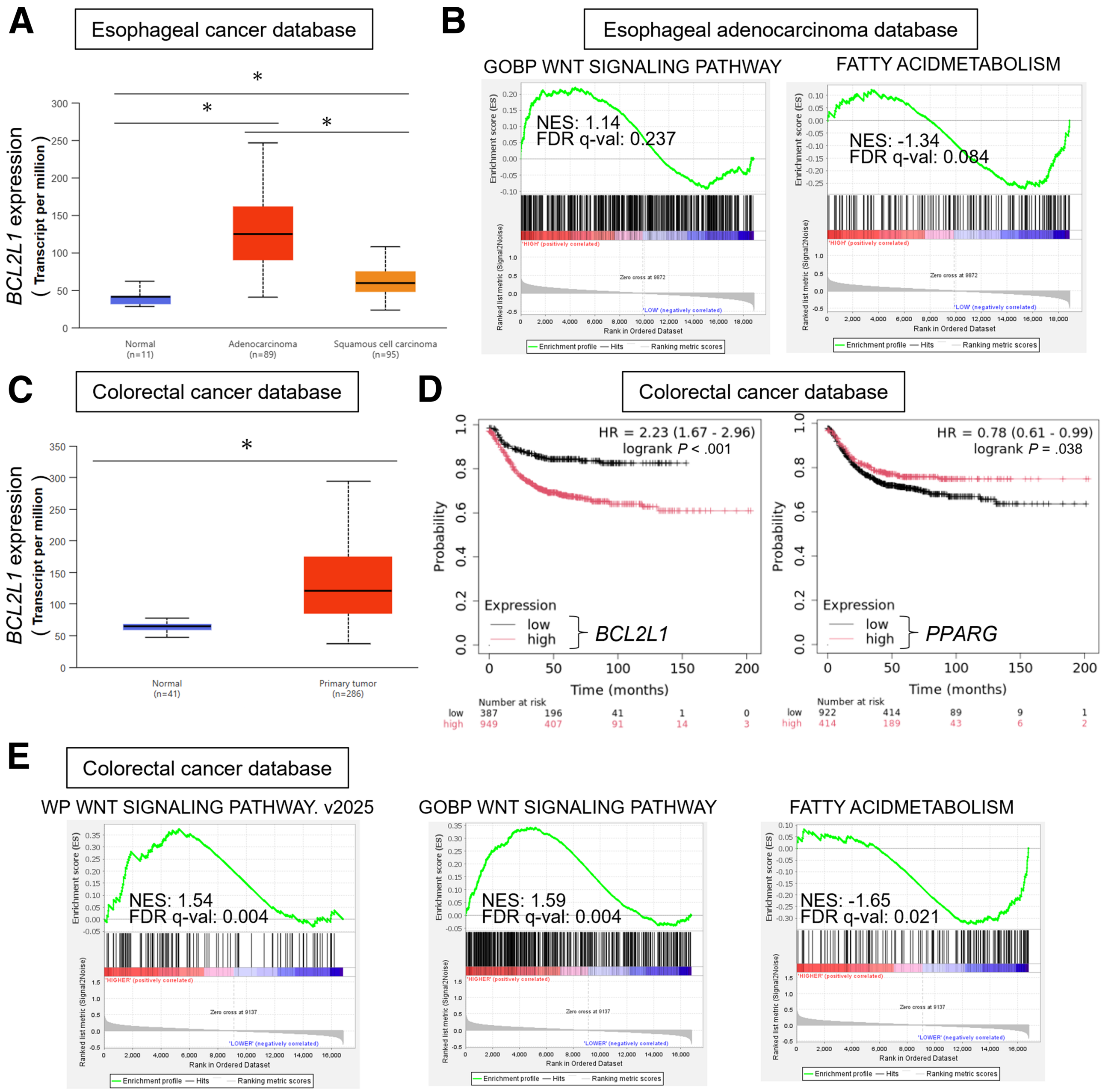

Figure 14. Analysis of Bcl-xL/BCL2L1 and PPARγ expression in human esophageal and colorectal cancer database. (*A*) Box plot showing the *BCL2L1* expression comparing the normal esophagus (n = 11), esophageal adenocarcinoma (n = 89) and esophageal squamous cell carcinoma (n = 95) based on the TCGA database. The *P* value was calculated using a *t* test. (*B*) Sigmoid curve showing the result of the GSEA with gene sets for WNT target genes and fatty acid metabolism, comparing between high- and low- *BCL2L1* expressed esophageal adenocarcinoma based on the TCGA database (n = 44 vs 43). (*C*) Box plot showing the *BCL2L1* expression comparing the normal stomach (n = 41) and primary colorectal cancer tissue (n = 286) based on the TCGA database. The *P* value was calculated using a *t* test. (*D*) Survival analysis of patients with GC (n = 1336) stratified by high vs low *BCL2L1* and high vs low *PPARG*. The *P* value was calculated using a log-rank test. (*E*) Sigmoid curve showing the result of the GSEA with gene sets for WNT target genes and fatty acid metabolism, comparing between high- and low- *BCL2L1* expressed colorectal cancer based on the TCGA database (n = 112 vs 112). Mean ± standard error of the mean. *$P < .05$.

compromises proliferation. Moreover, external Wnt and R-spondin supplementation were included to maintain optimal culture conditions. APC-deficient organoids can still respond quantitatively to extracellular Wnt modulation, and signaling output is influenced by multiple layers of regulation beyond APC loss alone.

In summary, our study identifies Bcl-xL as a multifaceted regulator of gastric tumorigenesis that integrates stem cell

Table 1. The Association Between GC Development and Use of Pioglitazone in the Patients With Diabetes Mellitus

Factors	GC (n = 1378)	No GC (n = 28,527)	Crude OR (95% CI)	Adjusted OR[a] (95% CI)	*P*
Female	313 (2.90)	10,492 (97.10)	1	1	
Male	1065 (5.58)	18,035 (94.42)	1.98 (1.74–2.25)	1.70 (1.48–1.96)	<.0001[b]
Age <70 *y*	378 (3.32)	10,996 (96.68)	1	1	
Age >70 *y*	1000 (5.40)	17,531 (94.60)	1.66 (1.47–1.87)	1.70 (1.50–1.92)	<.0001[b]
Nonsmoking	635 (3.68)	16,617 (96.32)	1	1	
Smoking	743 (5.87)	11,910 (94.31)	1.63 (1.47–1.82)	1.39 (1.24–1.57)	<.0001[b]
No *H pylori* eradication	1262 (4.31)	28,021 (95.69)	1	1	
H pylori eradication	116 (18.05)	506 (81.57)	5.10 (4.13–6.28)	5.23 (4.23–6.48)	<.0001[b]
Charlson comorbidity index	3.38 ± 2.49	2.72 ± 2.13	–	–	–
No pioglitazone	1325 (4.64)	27,251 (95.36)	1	1	
Pioglitazone	53 (3.99)	1276 (96.01)	0.85 (0.65–1.13)	0.74 (0.56–0.99)	.0419[b]
No metformin	1162 (4.69)	23,623 (95.31)	1	1	
Metformin	216 (4.22)	4904 (95.78)	0.90 (0.77–1.04)	0.91 (0.79–0.86)	.267

CI, confidence interval; GC, gastric cancer; OR, odds ratio.
[a]OR adjusted for age >70 years, sex, smoking, *H pylori* eradication, and Charlson comorbidity index.
[b]*P* < .05.

signaling, transcriptional control, and metabolic regulation. These findings not only redefine the biological functions of Bcl-xL in GC but also highlight new therapeutic opportunities. Targeting Bcl-xL–dependent signaling networks, either directly or through modulation of downstream pathways such as Wnt or PPARγ, may represent a promising strategy for the prevention and treatment of GC.

Materials and Methods

Mice

Mist1-CreERT, *Apc*$^{flox/flox}$, and LoxP-stop-LoxP (LSL)-*Trp53*^{R172H} mice were provided by Columbia University, and *bcl-xl*$^{flox/flox}$ mice were provided by Osaka University. LSL-TdTomato and LSL-*Kras*^{G12D} mice were purchased from Jackson laboratory. *Lgr5*-DTR-EGFP mice were obtained from Genentech (South San Francisco, CA).[68] WT and nude mice were purchased from Crea Japan. Mice other than nude mice were from the C57BL6J background. All mice were maintained in a specific pathogen-free facility at the University of Tokyo in accordance with the institutional guidelines for animal experimentation and were provided with commercially available food and water ad libitum. All animal studies and procedures were approved by the ethics committee of the University of Tokyo (ID: 24-2). Both male and female mice were used in this study. No obvious sex-based differences in phenotype were observed. For each experiment, animals were sex- and age-matched between experimental groups.

For analyses using mouse gastric tumor models, WT, *Mist1*-CreERT; *bcl-xl*$^{flox/flox}$, *Mist1*-CreERT; *Apc*$^{flox/flox}$, and *Mist1*-CreERT; *Apc*$^{flox/flox}$; *bcl-xl*$^{flox/flox}$ mice were used. When mice were incorporated LSL-TdTomato, the genetically modified proteins were labeled with fluorescence. Lineage tracing was induced by administering TAM at 6 mg per 20 g body weight on 2 separate days, with a 1-day interval between injections to all 6-week-old mice. Thus, mice received a total of 2 TAM injections, and they were sacrificed at 2 months for analysis. For analysis using the organoid culture system, transgenic organoids were established from WT, *Apc*$^{flox/flox}$, LSL-*Kras*^{G12D}; LSL-*Trp53*^{R172H}; *Apc*$^{flox/flox}$, and LSL-*Kras*^{G12D}; LSL-*Trp53*^{R172H}; *Apc*$^{flox/flox}$; *bcl-xl*$^{flox/flox}$ mice.

Organoid Culture

Organoids were generated as described previously.[22–24] Mice were cervically dislocated, the gastric antrum was excised and washed with phosphate-buffered saline (PBS), then shaken in 5 mM EDTA (Invitrogen) + PBS for 45 minutes at 4 °C. Cells derived from the gastric mucosa were detached from the serosa; the required amount of cells were collected, dissolved in Matrigel (Corning), seeded in 24-well plates, and medium was added 30 minutes later. The medium was a Wnt3a and R-spondin 3 conditioned medium[69] prepared from L-WRN cells, which are modified L-Wnt3a cells derived from mouse L cells, and advanced DMEM and F12 (Invitrogen) mixed 2:1. It was then mixed with penicillin and streptomycin (Wako), Gluta MAX (Invitrogen), HEPES (Invitrogen), B27 (Invitrogen), N2 (Invitrogen), N-acetylcysteine (Wako, 1 mM), and Nicotinamide (Sigma-Aldrich, 10 mM). Epidermal growth factor (Wako, 50 ng/mL), fibroblast growth factor 10 (Peprotech, 100 ng/mL), Y-27632 (Wako, 10 μM), Gastrin (Tocris, 0.01 μM), and Primocin (Invitrogen) were used in the mixture. The medium was changed twice a week. For the quantification of organoid diameters, n = 10 refers to 10 organoids randomly selected from the same condition.

Treatment In Vitro

For the pharmacologic inhibition of Bcl-xL, A-1155463 (Selleck: S7800) was used. A-1155463 is a highly potent and selective Bcl-xL inhibitor that exhibits picomolar

Table 2. Primer Sequences Used for Quantitative PCR

Targets	Forward or reverse	Sequences
Lgr5	Forward	5'-CCACAGCAACAACATCAGGT-3'
	Reverse	5'-AACAAATTGGATGGGGTTGT-3'
Lgr4	Forward	5'-CCTTCACCCAAGCACTGGATA-3'
	Reverse	5'-AGGTCGTTACCAGCCAGTTG-3'
Axin2	Forward	5'-TGCATCTCTCTCTGGAGCTG-3'
	Reverse	ACTGACCGACGATTCCATGT-3'
Sox9	Forward	5'-GAGGCCACGGAACAGACTCA-3'
	Reverse	5'-CAGCGCCTTGAAGATAGCATT-3'
Cd44	Forward	5'-ACAGTACCTTACCCACCATG-3'
	Reverse	5'-GGATGAATCCTCGGAATTAC-3'
Cmyc	Forward	5'-GAGCTGTTTGAAGGCTGGATTT-3'
	Reverse	5'-TCCTGTTGGTGAAGTTCACGTT-3'
Fzd7	Forward	5'-TGCAGTCATGGCGTCGCTTCTA-3'
	Reverse	5'-TTCGGCCACTGCCTTTGCCTTT-3'
Fzd5	Forward	5'-ATCCTCCGAGAGTTCTGTCCTTGG-3'
	Reverse	5'-TCGTCTCCTTCTTCCCTTTGCCT-3'
Ccnd1	Forward	5'-CGTGGCCTCTAAGATGAAGGA-3'
	Reverse	5'-TCGGGCCGGATAGAGTTGT-3'
Mist1	Forward	5'-GTTTTCCCAGGAGGTGGTG-3'
	Reverse	5'-AAGCTACGTGTCCTTGTCCC-3'
Bcl2l1	Forward	5'-GTGGAAAGCGTAGACAAGGA-3'
(*Bclxl*)	Reverse	5'-GTTCCCGTAGAGATCCACAA-3'
Bcl2	Forward	5'-ATGGGGTGAACTGGGGGAGGATTG-3'
	Reverse	5'-GGCCAGGCTGAGCAGGGTCTTC-3'
Bax	Forward	5'-TTGCTGATGGCAACTTCAAC-3'
	Reverse	5'-GATCAGCTCGGGCACTTTAG-3'
Bak	Forward	5'-AGCCGGGAATGCCTACGAAC-3'
	Reverse	5'-GGCCCAACAGAACCACACCA-3'
Pparg	Forward	5'-GGAGCCTAAGTTTGAGTTTGCTGTG-3'
	Reverse	5'-TGCAGCAGGTTGTCTTGGATG-3'
Gapdh	Forward	5'-AGAAACCTGCCAAGTATGATGAC-3'
	Reverse	5'-TCATTGTCATACCAGGAAATGAG-3'

binding affinity for Bcl-xL, while showing more than 1000-fold weaker binding affinity for Bcl-2 and related proteins, including Bcl-w (Ki = 19 nM) and Mcl-1 (Ki > 440 nM), as described in the manufacturer's documentation.

A-1155463 (5 mg) was dissolved in 0.7465 mL of DMSO to prepare a 10-mM stock solution, which was further diluted to generate working solutions at the indicated concentrations. During organoid passaging, the Bcl-xL inhibitor at each concentration was added to the culture medium. After 72 hours, organoids were passaged again. Starting the day after passaging, organoid images were captured, and the long-axis diameter of each organoid was measured to compare changes in organoid growth over time.

Xenograft

Organoids are composed solely of epithelial cells and are not influenced by the tumor microenvironment, such as stromal or immune components. Therefore, xenograft models are essential for evaluating the interaction between tumor cells and their microenvironment.[49] Organoid cells (1×10^6 cells) were added to 100 μL of Matrigel and 100 μL of advanced DMEM and F12 and injected subcutaneously into the back of nude mice at 6 weeks after birth with a 26 G needle. The tumor diameters of the subcutaneous tumors were measured, and the volumes were calculated every week. After 1 month, the mice were sacrificed, and the subcutaneous tumors were removed for analysis.

Hematoxylin and Eosin Stain

Hematoxylin and eosin staining was performed on paraffin-embedded sections of mouse tissues and organoids fixed in 10% buffered formalin solution (pH 7.4). After deparaffinization and rinsing, nuclear staining was performed with hematoxylin. After rinsing in running water, the sections were stained with eosin, dehydrated, permeabilized, sealed with Mount Quick (DAI), and the specimens were examined.

Immunohistochemistry

Immunohistochemistry was performed on formalin-fixed and paraffin-embedded sections of mouse tissue. The sections were deparaffinized, autoclaved at 120 °C for 10 minutes for antigen activation, soaked in 3% hydrogen peroxide for 5 minutes for inactivation of endogenous peroxidase, and blocked in 10% goat serum for 30 minutes. The sample was outlined with a water-repellent pen and reacted with primary antibody diluted in PBS mixed with 2.5% Bovine Serum Albumin Cohn Fraction V (hereafter referred to as 2.5% BSA) and allowed to stand overnight at 4 °C. The next day, the antibody was washed with PBS, followed by the reaction of the secondary antibody, and allowed to stand at 4 °C for 1 hour. After washing with PBS, the antibody was incubated with avidin-biotin–labeled enzyme complex (VECTASTAIN Elite ABC Kit [Vector Laboratories]) for 20 minutes at room temperature, the avidin-biotin complex method was performed, and the reaction was carried out with enzyme substrate solution (Liquid 3,3'-diaminobenzidine [DAB] + Substrate Chromogen System, DAKO) for tens of seconds to several minutes, followed by rinsing. Nuclear staining was performed with hematoxylin, followed by rinsing, dehydration, permeabilization, sealing with MountQuick (DAI), and mirror examination. Bcl-xL (1:200, Cell Signaling Technology: 2764S), cyclinD1 (1:200, Thermo Fisher Scientific: RM-91004-SO), CD44v (1:200, BIO RAD: MCA1967), and cleaved caspase 3 (1:200, Cell Signaling Technology: 9661S) were used for immunohistochemistry.

Immunofluorescence

Fluorescent immunostaining was performed on formalin-fixed and paraffin-embedded sections of mouse tissues and organoids. The sections were deparaffinized and autoclaved at 120 °C for 10 minutes for antigen activation. The samples were surrounded with a pen for water generation, reacted with primary antibody diluted in 2.5%

BSA, and left overnight at 4 °C. After washing with PBS the next day, secondary antibodies (Alexa Fluor 555, Alexa Fluor 488, Alexa Fluor 647 [Invitrogen]) diluted 1:200 in 2.5% BSA were reacted and allowed to stand at room temperature for 1 hour. Fluoro-KEEPER Antifade Reagent, Non-Hardening Type with 4′,6-diamidino-2-phenylindole (Nacalai), and then the cells were stained, sealed, and microscopically examined. Bcl-xL (1:400, Cell Signaling Technology: 2764S), β-catenin (1:400, BD Biosciences: BD61053), Ki67 (1:500, Cell Signaling Technology: 12202S), Sox9 (1;400, Merck: AB5535), YAP (total-YAP; 1:200, Cell Signaling Technology: 14074), red fluorescent protein (1:400, ROCKLAND: 600401379), GFP (1:400, Abcam: ab13970), cyclinD1 (1:400, Thermo Fisher Scientific: RM-91004-SO), cleaved caspase 3 (1:200, Cell Signaling Technology: 9661S), CD44v (1:200, Bio RAD: MCA1967), E-cadherin (1:200, BD biosciences: 610181), AQP5 (1:200, ATLAS ANTIBODY, HPA065005), TROP2 (1:200, R and D, AF650), and PPARγ (1:400, Cell Signaling Technology: 2435) were used for immunohistochemistry.

In Situ Hybridization

In situ hybridization was performed on formalin-fixed, paraffin-embedded sections of mouse tissue using the RNAscope 2.5 HD Detection Kit BROWN (ACD). The process involved 60 minutes of baking at 60 °C, 2 5-minute dips in xylene, 100% soaked in ethanol twice for 5 minutes and dried for 5 minutes; then baked at 60 °C for 30 minutes, soaked in hydrogen peroxide solution for 10 minutes, then soaked in 1× Target Retrieval Buffer (ACD) and boiled at 100 °C for 15 minutes. The cells were washed with distilled water, dehydrated with 100% ethanol, soaked in 5 to 8 drops of hydrogen peroxide (ACD), allowed to stand at room temperature for 10 minutes, soaked in Protease Plus (ACD) for 30 minutes at 40 °C, washed with distilled water, and hybridized at 40 °C for 2 hours with *Lgr5* probe (ACD: 312171). The cells were washed in 1× wash buffer (ACD) for 2 minutes, immersed in 20× saline sodium citrate buffer (1000 mL distilled water with NaCl 175.3 g + sodium citrate 88.2 g adjusted to pH 7.0 and autoclaved sterile) and left overnight. After washing with 1× wash buffer for 2 minutes, AMP1 (ACD) was added dropwise to cover the area enclosed by the hydrophobic barrier pen and allowed to stand for 30 minutes at 40 °C. After washing with 1×wash buffer for 2 minutes, AMP2 (ACD) was added drop and allowed to stand for 15 minutes at 40 °C. After washing with 1× wash buffer for 2 minutes, AMP3 (ACD) was added drop and allowed to stand for 30 minutes at 40 °C. The cells were washed with 1× wash buffer for 2 minutes, AMP4 (ACD) was added drop and allowed to stand at 40 °C for 15 minutes. The cells were washed with 1× wash buffer for 2 minutes, AMP5 (ACD) was added drop and allowed to stand at room temperature for 30 minutes. The cells were washed with 1× wash buffer for 2 minutes, AMP6 (ACD) was added drop and allowed to stand at room temperature for 15 minutes. The cells were washed with 1× wash buffer for 2 minutes, then DAB-A and DAB-B (ACD) were added dropwise at 1:1 and allowed to stand for 10 minutes at room temperature. After rinsing, nuclear staining was performed with hematoxylin, soaked in 0.02% ammonia water, rinsed, soaked in 70% ethanol twice for 5 minutes, soaked in 100% ethanol twice for 5 minutes, dehydrated, permeated with xylene twice for 5 minutes, sealed with Mount Quick (DAI), and specimens were examined.

Histopathologic Analysis

All images were captured with a Leica DMi8 inverted fluorescence microscope and manually quantified after visualization using LAS X software. For each mouse, the average value was calculated from 3 randomly selected, well-oriented fields at 10× magnification. Quantification was conducted manually on fluorescence or chromogenic images by investigators blinded to the experimental conditions. Group-level values were calculated by averaging across animals and are presented as mean ± standard error of the mean. Unless otherwise indicated, analyses were performed using 3 to 5 biologically independent mice per group.

Western Blotting

Gastric tissue and organoid cells were homogenized by adding cell lysate buffer (50 mM Tris HCl, 1% Triton-X, 5 mM EDTA, 1 mM Na3VO4, 1.25 mM NaF, protease inhibitor cocktail (cOmplete Mini, Roche) and centrifuged at 12,000 g for 5 minutes to extract proteins from the supernatant. Proteins were separated by polyacrylamide gel electrophoresis and transferred to polyvinylidene fluoride membrane (Pall Corporation). The cells were blocked by shaking with 5% milk for 1 hour, washed with Tris Buffered Saline with Tween 20 (TBS-T), and reacted with the primary antibody by shaking overnight at 4 °C. The reaction was repeated at 4 °C for 1 hour. After washing with TBS-T, the antibody was reacted with horseradish peroxidase–conjugated secondary antibody (GE Healthcare) while shaking at room temperature for 1 hour. After washing with TBS-T, proteins were detected by exposure with a chemiluminescence detection system using the luminescent reagent ImmunoStar LD (Fujifilm Wako Pure Chemicals). Bcl-xL (1:1000, Cell Signaling Technology: 2764S), cyclinD1 (1:1000, Thermo Fisher Scientific: RM-91004-SO), PPARγ (1:1000, Cell Signaling Technology: 2435), total caspase-3 (1:1000, Cell Signaling Technology: 9662), cleaved caspase-3 (1:1000, Cell Signaling Technology: 9664), and β-actin (1:10,000, cell signaling technology: 5125S) antibodies were used for Western blotting.

Real-Time Polymerase Chain Reaction

RNA extraction from gastric tissue was performed using NucleoSpin RNA (Macherey-Nagel), and RNA extraction from organoids was performed using RNeasy Micro kit (Qiagen). cDNA was synthesized from RNA using ImProm-ll Reverse Transcription System (Promega). Amplification of cDNA was performed using StepOnePlus Real Time PCR System Applied Biosystems and FastStart Universal SYBR Green Master (Rox) (Roche). The results obtained were

standardized and analyzed with glyceraldehyde-3-phosphate dehydrogenase. A list of primers used is given in Table 2.

Lentivirus Transduction

Organoids in culture were washed with PBS and recovered from Matrigel (Corning) basement membrane matrix using Cell Recovery Solution (Corning). We collected single-celled organoids using TrypLE Express (Thermo Fisher Scientific), added pLV-EF1-Cre-2a-vsfGFP-2a-puro lentivirus (originated in Tokyo University[24]) at an optimized concentration for infection and the above medium (after adding a solution of 60 μL) and Trans Dux MAX (System Biosciences, 0.6 μL), centrifuged at 300 g for 30 minutes and mixed well with a pipette 3 times, dissolved in Matrigel, seeded in 24-well plates, and added medium 60 minutes later. The next day, the virus infection was confirmed by microscopic observation of GFP expression. Two to 3 days later, puromycin was added to the medium at 0.5 to 10 μg/mL to select for organoids undergoing genetic recombination. Other methods of selection included culture in medium without Wnt3A conditioned medium or R-spondin 3 conditioned medium, or by combining the method of collecting and culturing only organoids expressing GFP by genetic recombination under a microscope. We were unable to establish stable knockout organoids from either *bcl-xL*$^{flox/flox}$ mice or *Apc*$^{flox/flox}$; *bcl-xL*$^{flox/flox}$ mice following Cre-expressing lentivirus-mediated recombination, despite repeated attempts ($n > 5$). To analyze the cell proliferation rate of organoids, organoids of similar initial size ($n = 10$) were selected on day 1, and longitudinal changes in length diameter were measured using tile-scan imaging.

Adenovirus Infection

Adenoviruses were purchased in vectorbuilder (VB210912-1088ebt: pAV[Exp]-EF1A>mPparg[NM_011146.3](ns):T2A:DsRed_Express2 and VB220822-1173ydv: pAV[Exp]-CAG>mHnf4a[NM_008261.3]:IRES:mKate2) and were suspended in organoid cells, arranged for multiplicity of infection of 100, after dissolution in Matrigel. After 3 hours, organoids were seeded in 24-well plates. After 48 hours, the medium was replaced with medium without growth factors. Organoids without the selection procedure were harvested after 24 hours, and their diameters were calculated for proliferation analysis in 5 days.

The gene expression was analyzed by Western blotting, qPCR, and RNA sequencing.

RNA Sequencing

Sequences were performed on RNA extracted from organoids using the NovaSeq 6000 system from Illumina. Global normalization was performed on the obtained read information using TopHat-2.0.13 bowtie 2-2.2.3 as the alignment tool and Subio Platform as the analysis tool. Variability multipliers greater than 1.7 and *P* values less than .05 after correction for multiple testing using the Benjamini–Hochberg method were counted as significant variable genes. Gene Ontology analysis and Kyoto Encyclopedia of Genes and Genomes pathway analysis were performed using the Database for Annotation, Visualization, and Integrated Discovery. GSEA was also performed to analyze gene groups with significant expression variation. A false discovery rate threshold of 0.25 was used to define statistical significance. However, to illustrate similarities between several models, sigmoid curves were plotted to visualize overall trends, including gene sets that did not reach statistical significance.

Data Analysis of Human Gastric Cancer, Esophageal Cancer, and Colorectal Cancer

First, the expression status of Bcl-xL was evaluated by fluorescent immunostaining using human GC tissue microarrays. The tissue microarrays were purchased from US Biomax, and a total of 72 cases were scored according to the intensity of Bcl-xL expression and compared at each stage.

Second, using the UALCAN application and the TCGA database,[53] we evaluated *BCL2L1* expression in normal gastric mucosa and GC, esophageal cancer, and colorectal cancer.

Third, RNA sequence data of 450 GC cases registered in TCGA were analyzed using the web tool "Phantasus" (https://artyomovlab.wustl.edu/phantasus/),[55] and the expression status of *BCL2L1* was compared with WNT target genes.

Fourth, RNA sequence data of 412 GC (PanCancer Atlas), 87 esophageal adenocarcinoma (PanCancer Atlas), and 224 colorectal cancer[70] cases registered in TCGA were downloaded via Cbioportal website (https://www.cbioportal.org/) and analyzed with GSEA.

Fifth, we used the Kaplan-Meier Plotter (http://kmplot.com/analysis/index.php?p=service&cancer=gastric), a web tool for analyzing the gene expression database and the prognosis of patients with GC and colorectal cancer, to examine the contribution of each gene to survival of 875 patients with GC and 1336 patients with colorectal cancer.

Finally, we compared the occurrence of GC with gender, age, smoking, eradication of *H pylori*, comorbidity (Charlson comorbidity index), pioglitazone and metformin status in 29,905 hospitalized diabetic patients registered in the DPC of the University of Tokyo Hospital from April 2014 to March 2019, as described previously.[71–76] The ethics review number was 2058-[2]. Regarding the codes used to identify the disease, diabetes mellitus were E100, E101, E106, E108–E111, E116, E118–E121, E126, E128–E131, E136, E138 –E141, E146, E148, E149, E102–E105, E107, E112–E117, E122–E125, E132–E135, E137 E142–E145, and E147, and gastric cancers were C160, C161, C162, C163, C164, C165, C166, C168, and C169. Regarding the drug codes, pioglitazone was 610432040, 610432041, 621990901, 621991001, 621986301, 621986401, 622048401, 622048501, 622086001, 622086101 and metformin was 620004480, 620005570, 621676001, 621 974701, 621986301, 621986401, 622242501, 622412701, 622417101, 622417201, 622421101, 622421201, 6224

21901, 6224 622422001, 622424401, 622424501, 6224 27201, 622427301, 622432601, 622432701, 622436301, 622438401, 622438501, 622448601, 622450301, 6224 50401, 6224 622466601, 622517101, and 622654401. Crude OR and adjusted OR (adjustment factors were age, gender, Charlson comorbidity index, smoking, and *H pylori* eradication) were calculated using SAS as statistical software, and $P < .05$ was considered significant.

The Analysis of Single-Cell RNA Sequencing

Single-cell RNA sequencing data of mice GC samples and patients with GC were obtained from GSE217498 and GSE239676, respectively. Filtered count matrices were imported to R for further analysis using the Seurat package. Dimensionality reduction was performed by PCA and Uniform Manifold Approximation and Projection embedding with 30 principal components and resolution 1.0. Epithelial cells were then subsetted based on epithelial markers. For the human dataset, *BCL2L1* expression in tumor epithelial cells was obtained using the "AverageExpression" function in Seurat. Patients were then classified into *BCL2L1*-high or *BCL2L1*-low. Then we conducted differential expression and GSEA on pseudobulk workflow by using "AggregateExpression" function in the Seurat and R packages DESeq2 and fgsea.

Quantification and Statistical Analysis

Continuous variables are expressed as a means with 95% standard deviations, whereas categorical variables are expressed as numbers and frequencies (%). The differences between the means were compared using either the Student *t* test or the Wilcoxon test. Statistical significance was set at $P < .05$.

Supplementary Material

Note: To access the supplementary material accompanying this article, visit the full text version at https://doi.org/10.1016/j.jcmgh.2026.101880.

References

1. Arai J, Niikura R, Hayakawa Y, et al. Gastric cancer incidence after helicobacter pylori eradication and lifestyle-related risk factors: a population-based cohort study. Helicobacter 2026;31:e70138.
2. Bray F, Laversanne M, Sung H, et al. Global cancer statistics 2022: GLOBOCAN estimates of incidence and mortality worldwide for 36 cancers in 185 countries. CA Cancer J Clin 2024;74:229–263.
3. Kankeu Fonkoua L, Yee NS. Molecular characterization of gastric carcinoma: therapeutic implications for biomarkers and targets. Biomedicines 2018;6.
4. Ansari S, Gantuya B, Tuan VP, et al. Diffuse gastric cancer: a summary of analogous contributing factors for its molecular pathogenicity. Int J Mol Sci 2018;19.
5. Kakiuchi M, Nishizawa T, Ueda H, et al. Recurrent gain-of-function mutations of RHOA in diffuse-type gastric carcinoma. Nat Genet 2014;46:583–587.
6. Ghatak S, Chakraborty P, Sarkar SR, et al. Novel APC gene mutations associated with protein alteration in diffuse type gastric cancer. BMC Med Genet 2017;18:61.
7. Kohno Y, Yamamoto H, Hirahashi M, et al. Reduced MUTYH, MTH1, and OGG1 expression and TP53 mutation in diffuse-type adenocarcinoma of gastric cardia. Hum Pathol 2016;52:145–152.
8. Comprehensive molecular characterization of gastric adenocarcinoma. Nature 2014;513:202–209.
9. Chen Q, Weng K, Lin M, et al. SOX9 modulates the transformation of gastric stem cells through biased symmetric cell division. Gastroenterology 2023;164:1119–1136.e12.
10. Flanagan DJ, Barker N, Nowell C, et al. Loss of the Wnt receptor frizzled 7 in the mouse gastric epithelium is deleterious and triggers rapid repopulation in vivo. Dis Model Mech 2017;10:971–980.
11. Sigal M, Logan CY, Kapalczynska M, et al. Stromal R-spondin orchestrates gastric epithelial stem cells and gland homeostasis. Nature 2017;548:451–455.
12. Chang W, Wang H, Kim W, et al. Hormonal suppression of stem cells inhibits symmetric cell division and gastric tumorigenesis. Cell Stem Cell 2020;26:739–754.e8.
13. Hayakawa Y, Jin G, Wang H, et al. CCK2R identifies and regulates gastric antral stem cell states and carcinogenesis. Gut 2015;64:544–553.
14. Hayakawa Y, Nakagawa H, Rustgi AK, et al. Stem cells and origins of cancer in the upper gastrointestinal tract. Cell Stem Cell 2021;28:1343–1361.
15. Matsuo J, Douchi D, Myint K, et al. Iqgap3-ras axis drives stem cell proliferation in the stomach corpus during homoeostasis and repair. Gut 2021;70:1833–1846.
16. Arnold K, Sarkar A, Yram MA, et al. Sox2(+) adult stem and progenitor cells are important for tissue regeneration and survival of mice. Cell Stem Cell 2011;9:317–329.
17. Han S, Fink J, Jörg DJ, et al. Defining the identity and dynamics of adult gastric isthmus stem cells. Cell Stem Cell 2019;25:342–356.e7.
18. Hata M, Kinoshita H, Hayakawa Y, et al. GPR30-expressing gastric chief cells do not dedifferentiate but are eliminated via PDK-dependent cell competition during development of metaplasia. Gastroenterology 2020;158:1650–1666.e15.
19. Kinoshita H, Hayakawa Y, Niu Z, et al. Mature gastric chief cells are not required for the development of metaplasia. Am J Physiol Gastrointest Liver Physiol 2018;314:G583–G596.
20. Barker N, Huch M, Kujala P, et al. Lgr5(+ve) stem cells drive self-renewal in the stomach and build long-lived gastric units in vitro. Cell Stem Cell 2010;6:25–36.
21. Zhu L, Gibson P, Currle DS, et al. Prominin 1 marks intestinal stem cells that are susceptible to neoplastic transformation. Nature 2009;457:603–607.
22. Hayakawa Y, Ariyama H, Stancikova J, et al. Mist1 expressing gastric stem cells maintain the normal and neoplastic gastric epithelium and are supported by a perivascular stem cell niche. Cancer Cell 2015;28:800–814.

23. Hayakawa Y, Sakitani K, Konishi M, et al. Nerve growth factor promotes gastric tumorigenesis through aberrant cholinergic signaling. Cancer Cell 2017;31:21–34.
24. Arai J, Hayakawa Y, Suzuki N, et al. ARID1A mutation drives gastric tumorigenesis via activating type 2 immune dominant microenvironment. iScience 2025;28:113117.
25. Arai J, Hata M, Ochiai Y, et al. Electroporation-induced gastric cancer models through epithelial-specific genetic modifications. Gastro Hep Adv 2026;5:100916.
26. Oya Y, Hayakawa Y, Koike K. Tumor microenvironment in gastric cancers. Cancer Sci 2020;111:2696–2707.
27. Arai J, Hayakawa Y, Tateno H, et al. Impaired glycosylation of gastric mucins drives gastric tumorigenesis and serves as a novel therapeutic target. Gastroenterology 2024;167:505–521.e19.
28. Niikura R, Hayakawa Y, Nagata N, et al. Non-helicobacter pylori gastric microbiome modulates prooncogenic responses and is associated with gastric cancer risk. Gastro Hep Adv 2023;2:684–700.
29. Sheng W, Malagola E, Nienhüser H, et al. Hypergastrinemia expands gastric ECL cells through CCK2R+ progenitor cells via ERK activation. Cell Mol Gastroenterol Hepatol 2020;10:434–449.
30. Arai J, Hayakawa Y, Tateno H, et al. The role of gastric mucins and mucin-related glycans in gastric cancers. Cancer Sci 2024;115:2853–2861.
31. Regimbald-Dumas Y, He X. Wnt signalling: What The X@# is WTX? EMBO J 2011;30:1415–1417.
32. Chiurillo MA. Role of the Wnt/β-catenin pathway in gastric cancer: An in-depth literature review. World J Exp Med 2015;5:84–102.
33. MacLeod RJ. MDR-1, Bcl-xL, H. pylori, and Wnt/β-catenin signalling in the adult stomach: how much is too much? Lab Invest 2012;92:1670–1673.
34. Chen YB, Aon MA, Hsu YT, et al. Bcl-xL regulates mitochondrial energetics by stabilizing the inner membrane potential. J Cell Biol 2011;195:263–276.
35. Bertini I, Chevance S, Del Conte R, et al. The anti-apoptotic Bcl-x(L) protein, a new piece in the puzzle of cytochrome c interactome. PLoS One 2011;6:e18329.
36. Yi CH, Pan H, Seebacher J, et al. Metabolic regulation of protein N-alpha-acetylation by Bcl-xL promotes cell survival. Cell 2011;146:607–620.
37. Li M, Wang D, He J, et al. Bcl-X(L): A multifunctional anti-apoptotic protein. Pharmacol Res 2020;151:104547.
38. Zhou WH, Tang F, Xu J, et al. Low expression of Beclin 1, associated with high Bcl-xL, predicts a malignant phenotype and poor prognosis of gastric cancer. Autophagy 2012;8:389–400.
39. Park HA, Broman K, Stumpf A, et al. Nutritional regulators of Bcl-xL in the brain. Molecules 2018;23.
40. Ikezawa K, Hikita H, Shigekawa M, et al. Increased Bcl-xL expression in pancreatic neoplasia promotes carcinogenesis by inhibiting senescence and apoptosis. Cell Mol Gastroenterol Hepatol 2017;4:185–200.e1.
41. Scherr AL, Mock A, Gdynia G, et al. Identification of BCL-XL as highly active survival factor and promising therapeutic target in colorectal cancer. Cell Death Dis 2020;11:875.
42. Ramesh P, Lannagan TRM, Jackstadt R, et al. BCL-XL is crucial for progression through the adenoma-to-carcinoma sequence of colorectal cancer. Cell Death Differ 2021;28:3282–3296.
43. Hikita H, Takehara T, Shimizu S, et al. The Bcl-xL inhibitor, ABT-737, efficiently induces apoptosis and suppresses growth of hepatoma cells in combination with sorafenib. Hepatology 2010;52:1310–1321.
44. Jin J, Xiong Y, Cen B. Bcl-2 and Bcl-xL mediate resistance to receptor tyrosine kinase-targeted therapy in lung and gastric cancer. Anticancer Drugs 2017;28:1141–1149.
45. Su CC. Tanshinone IIA inhibits human gastric carcinoma AGS cell growth by decreasing BiP, TCTP, Mcl-1 and Bcl-xL and increasing Bax and CHOP protein expression. Int J Mol Med 2014;34:1661–1668.
46. Yi H, Qiu MZ, Yuan L, et al. Bcl-2/Bcl-xl inhibitor APG-1252-M1 is a promising therapeutic strategy for gastric carcinoma. Cancer Med 2020;9:4197–4206.
47. Douchi D, Yamamura A, Matsuo J, et al. Induction of gastric cancer by successive oncogenic activation in the corpus. Gastroenterology 2021;161:1907–1923.e26.
48. Sakitani K, Hayakawa Y, Deng H, et al. CXCR4-expressing Mist1(+) progenitors in the gastric antrum contribute to gastric cancer development. Oncotarget 2017;8:111012–111025.
49. Kim M, Mun H, Sung CO, et al. Patient-derived lung cancer organoids as in vitro cancer models for therapeutic screening. Nat Commun 2019;10:3991.
50. Jones C, Avino M, Giroux V, et al. HNF4α acts as upstream functional regulator of intestinal Wnt3 and paneth cell fate. Cell Mol Gastroenterol Hepatol 2023;15:593–612.
51. O'Keefe RN, Carli ALE, Baloyan D, et al. A tuft cell - ILC2 signaling circuit provides therapeutic targets to inhibit gastric metaplasia and tumor development. Nat Commun 2023;14:6872.
52. González-García M, Pérez-Ballestero R, Ding L, et al. bcl-xLx is the major bcl-x mRNA form expressed during murine development and its product localizes to mitochondria. Development 1994;120:3033–3042.
53. Chandrashekar DS, Karthikeyan SK, Korla PK, et al. UALCAN: An update to the integrated cancer data analysis platform. Neoplasia 2022;25:18–27.
54. Xerri L, Parc P, Brousset P, et al. Predominant expression of the long isoform of Bcl-x (Bcl-xL) in human lymphomas. Br J Haematol 1996;92:900–906.
55. Kleverov M, Zenkova D, Kamenev V, et al. Phantasus, a web application for visual and interactive gene expression analysis. Elife 2024;13.
56. Cheng X, Dai E, Wu J, et al. Atlas of metastatic gastric cancer links ferroptosis to disease progression and immunotherapy response. Gastroenterology 2024;167:1345–1357.
57. Mukherjee N, Sheetz J, Shellman YG. Targeting the BCL2 family: advances and challenges in BH3 mimetic-based therapies. Int J Mol Sci 2025;26.

58. Semple RK, Chatterjee VK, O'Rahilly S. PPAR gamma and human metabolic disease. J Clin Invest 2006; 116:581–589.
59. Clevers H, Nusse R. Wnt/β-catenin signaling and disease. Cell 2012;149:1192–1205.
60. Clevers H. The intestinal crypt, a prototype stem cell compartment. Cell 2013;154:274–284.
61. Pate KT, Stringari C, Sprowl-Tanio S, et al. Wnt signaling directs a metabolic program of glycolysis and angiogenesis in colon cancer. EMBO J 2014;33:1454–1473.
62. Al-Mathkour M, Chen Z, Poveda J, et al. CDK1 drives SOX9-mediated chemotherapeutic resistance in gastric cancer. J Exp Clin Cancer Res 2025;44:284.
63. Scherr AL, Gdynia G, Salou M, et al. Bcl-xL is an oncogenic driver in colorectal cancer. Cell Death Dis 2016;7:e2342.
64. Choi S, Chen Z, Tang LH, et al. Bcl-xL promotes metastasis independent of its anti-apoptotic activity. Nat Commun 2016;7:10384.
65. Arai J, Niikura R, Hayakawa Y, et al. Autoimmune gastritis may be less susceptible to cancer development than Helicobacter pylori-related gastritis based on histological analysis. Gut 2024;73:1037–1038.
66. Arai J, Niikura R, Hayakawa Y, et al. Clinicopathological Features of Gastric Cancer with Autoimmune Gastritis. Biomedicines 2022;10.
67. Albuquerque C, Breukel C, van der Luijt R, et al. The 'just-right' signaling model: APC somatic mutations are selected based on a specific level of activation of the beta-catenin signaling cascade. Hum Mol Genet 2002; 11:1549–1560.
68. Tian H, Biehs B, Warming S, et al. A reserve stem cell population in small intestine renders Lgr5-positive cells dispensable. Nature 2011;478:255–259.
69. Miyoshi H, Stappenbeck TS. In vitro expansion and genetic modification of gastrointestinal stem cells in spheroid culture. Nat Protoc 2013;8:2471–2482.
70. Comprehensive molecular characterization of human colon and rectal cancer. Nature 2012;487:330–337.
71. Arai J, Niikura R, Hayakawa Y, et al. Use of antibiotics and probiotics reduces the risk of metachronous gastric cancer after endoscopic resection. Biology (Basel) 2021; 10.
72. Arai J, Niikura R, Hayakawa Y, et al. Nonsteroidal anti-inflammatory drugs prevent gastric cancer associated with the use of proton pump inhibitors after Helicobacter pylori eradication. JGH Open 2021;5:770–777.
73. Arai J, Suzuki N, Niikura R, et al. Chemoprevention for colorectal cancers: are chemopreventive effects different between left and right sided colorectal cancers? Dig Dis Sci 2022;67:5227–5238.
74. Arai J, Niikura R, Hayakawa Y, et al. Chemoprevention of oesophageal squamous-cell carcinoma and adenocarcinoma: a multicentre retrospective cohort study. Digestion 2022;103:192–204.
75. Arai J, Hayakawa Y, Niikura R, et al. Letter: potassium-competitive acid blockers may increase the risk of gastric cancer after helicobacter pylori eradication a retrospective multicentre-cohort analysis. Aliment Pharmacol Ther 2023;57:1196–1198.
76. Arai J, Niikura R, Hayakawa Y, et al. Association of probiotic use with nivolumab effectiveness against various cancers: a multicenter retrospective cohort study. Cancer Med 2023;12:16876–16880.

Received January 21, 2026. Accepted September 2, 2026.

Correspondence
Address correspondence to: Yoku Hayakawa, MD, PhD, Department of Gastroenterology, Graduate School of Medicine, The University of Tokyo, 7-3-1 Hongo, Tokyo, Japan. e-mail: yhayakawa-tky@umin.ac.jp.

Acknowledgments
The authors thank Editage for English language editing, supported by JSPS KAKENHI Grants-in-Aid for Scientific Research (no. 24K18531 [Junya Arai], 23H02744 [Yoku Hayakawa]).

CRediT Authorship Contributions

Yukiko Oya (Conceptualization: Equal; Data curation: Equal; Formal analysis: Lead; Investigation: Lead; Methodology: Lead; Writing – original draft: Equal)

Junya Arai, PhD, MD (Conceptualization: Equal; Data curation: Equal; Formal analysis: Equal; Investigation: Equal; Methodology: Equal; Writing – original draft: Equal)

Yoku Hayakawa (Conceptualization: Lead; Data curation: Lead; Formal analysis: Lead; Funding acquisition: Lead; Supervision: Lead; Writing – review & editing: Lead)

Toshiro Shiokawa (Formal analysis: Supporting; Writing – review & editing: Supporting)

Shintaro Shinohara (Formal analysis: Supporting; Writing – review & editing: Supporting)

Masahiro Hata (Formal analysis: Equal; Validation: Equal; Writing – review & editing: Equal)

Yuki Matsushita (Formal analysis: Equal; Writing – review & editing: Equal)

Hiroto Kinoshita (Formal analysis: Equal; Writing – review & editing: Equal)

Chie Uekura (Writing – review & editing: Equal)

Ken Kurokawa (Writing – review & editing: Equal)

Sohei Abe (Writing – review & editing: Equal)

Mayo Tsuboi (Writing – review & editing: Supporting)

Sozaburo Ihara (Writing – review & editing: Equal)

Nobumi Suzuki (Writing – review & editing: Equal)

Hayato Hikita (Writing – review & editing: Equal)

Tetsuo Takehara (Writing – review & editing: Equal)

Timothy C. Wang (Supervision: Equal; Writing – review & editing: Equal)

Mitsuhiro Fujishiro (Supervision: Equal; Writing – review & editing: Equal)

Conflicts of interest
The authors disclose no conflicts.

Funding
This study was supported by the Daiwa Securities Foundation, Kato Memorial Bioscience Foundation, UBE Foundation, Mochida Memorial Foundation for Medical and Pharmaceutical Research, Takeda Science Foundation, Kobayashi Foundation for Cancer Research, International Medical Research Foundation, and Cell Science Research Foundation (to Junya Arai), Yakult Bio-Science Foundation, Yasuda Memorial Foundation, Ono Pharmaceutical Foundation for Oncology, Immunology, and Neurology, Eli Lilly Japan KK Innovation Research Grant 2023, Research Grant of the Princess Takamatsu Cancer Research Fund, Suzuken Memorial Foundation, The Kurata Grants by The Hitachi Global Foundation, The Mitsubishi Foundation, AACR-Torrey Coast Foundation Transpacific Gastric and Esophageal Cancer Research Partnership Grant (to Yoku Hayakawa), JSPS KAKENHI Grants-in-Aid for Scientific Research (no. 24K18531 [Junya Arai], 23H02744, 24K22113, 26K02397 [Yoku Hayakawa], 23K07371 [Yukiko Oya]), P-CREATE/PROMOTE from AMED (Yoku Hayakawa), the Advanced Research and Development Programs for Medical Innovation (PRIME) (Yoku Hayakawa), JST FOREST Program (Yoku Hayakawa), and National Institutes of Health/National Cancer Institute funding including R01DK48077 and R35CA210088 (Timothy C. Wang).

Data Availability
Bulk RNA sequence data have been deposited at Zenodo at [https://doi.org/10.5281/zenodo.18188132] and are publicly available as of the date of publication. Further information and requests for resources and reagents should be directed to and will be fulfilled by the lead contact, Yoku Hayakawa (yhayakawa-tky@umin.ac.jp).

Cellular and Molecular Gastroenterology and Hepatology, Volume *21*

Supplemental information

B-Cell Lymphoma-Extra Large Promotes Gastric Tumorigenesis via Apoptosis-Independent Wnt–β-Catenin Activation and Peroxisome Proliferator–Activated Receptor γ Suppression

Yukiko Oya, Junya Arai, Yoku Hayakawa, Toshiro Shiokawa, Shintaro Shinohara, Masahiro Hata, Yuki Matsushita, Hiroto Kinoshita, Chie Uekura, Ken Kurokawa, Sohei Abe, Mayo Tsuboi, Sozaburo Ihara, Nobumi Suzuki, Hayato Hikita, Tetsuo Takehara, Timothy C. Wang, and Mitsuhiro Fujishiro

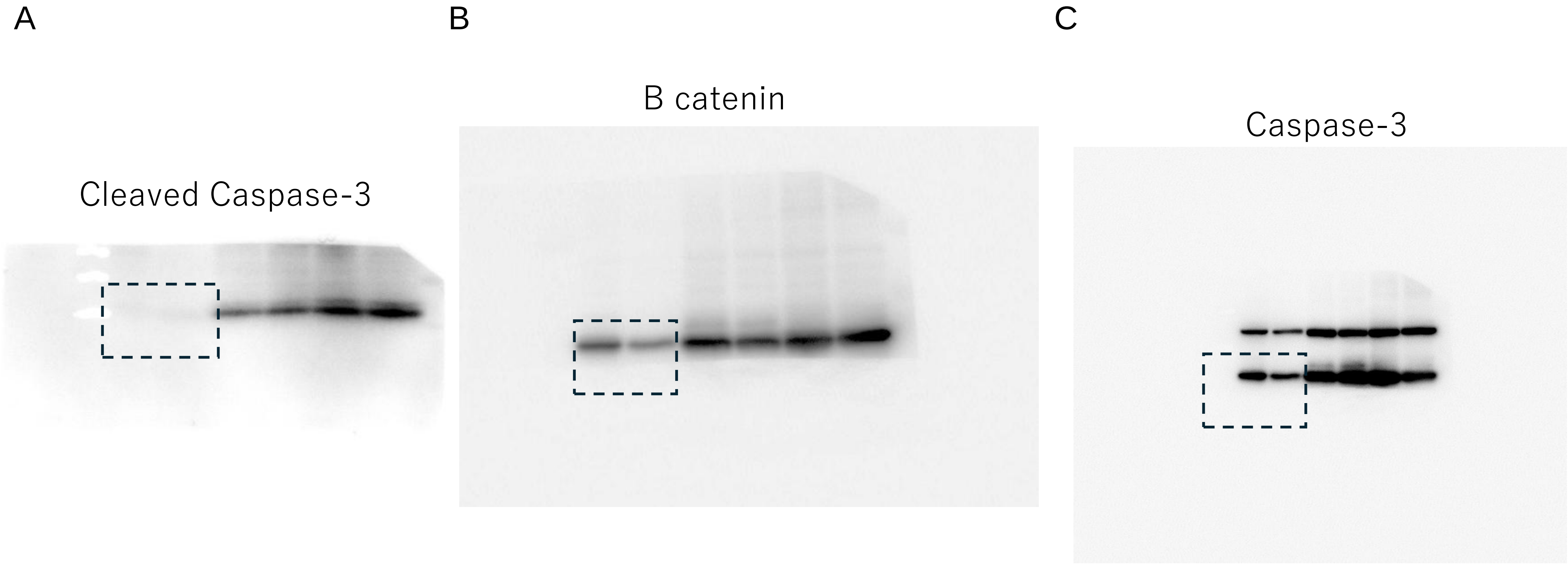
A
Cleaved Caspase-3
B
B catenin
C
Caspase-3

D

Bcl-xL

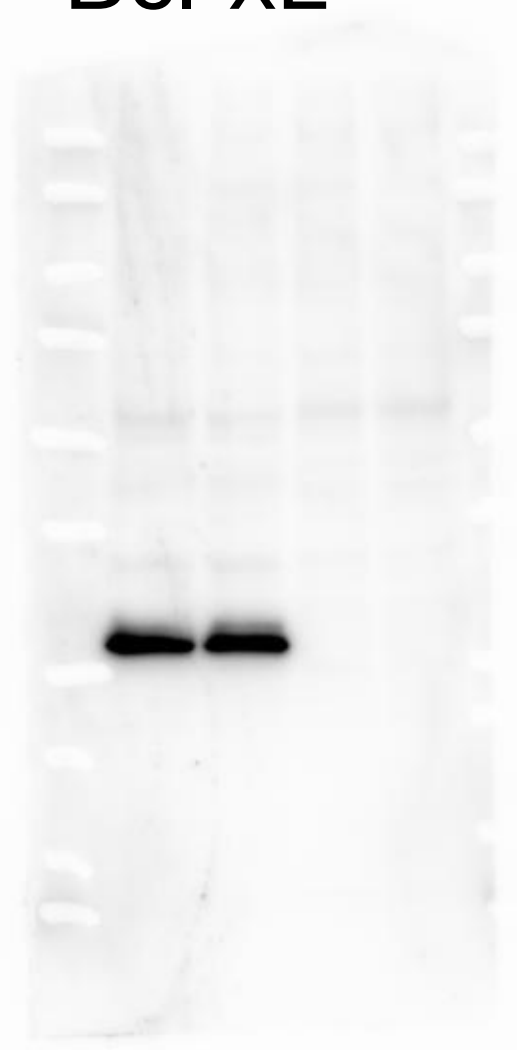

E

Cyclin D1

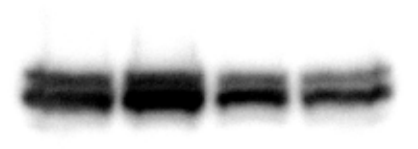

F

β-actin

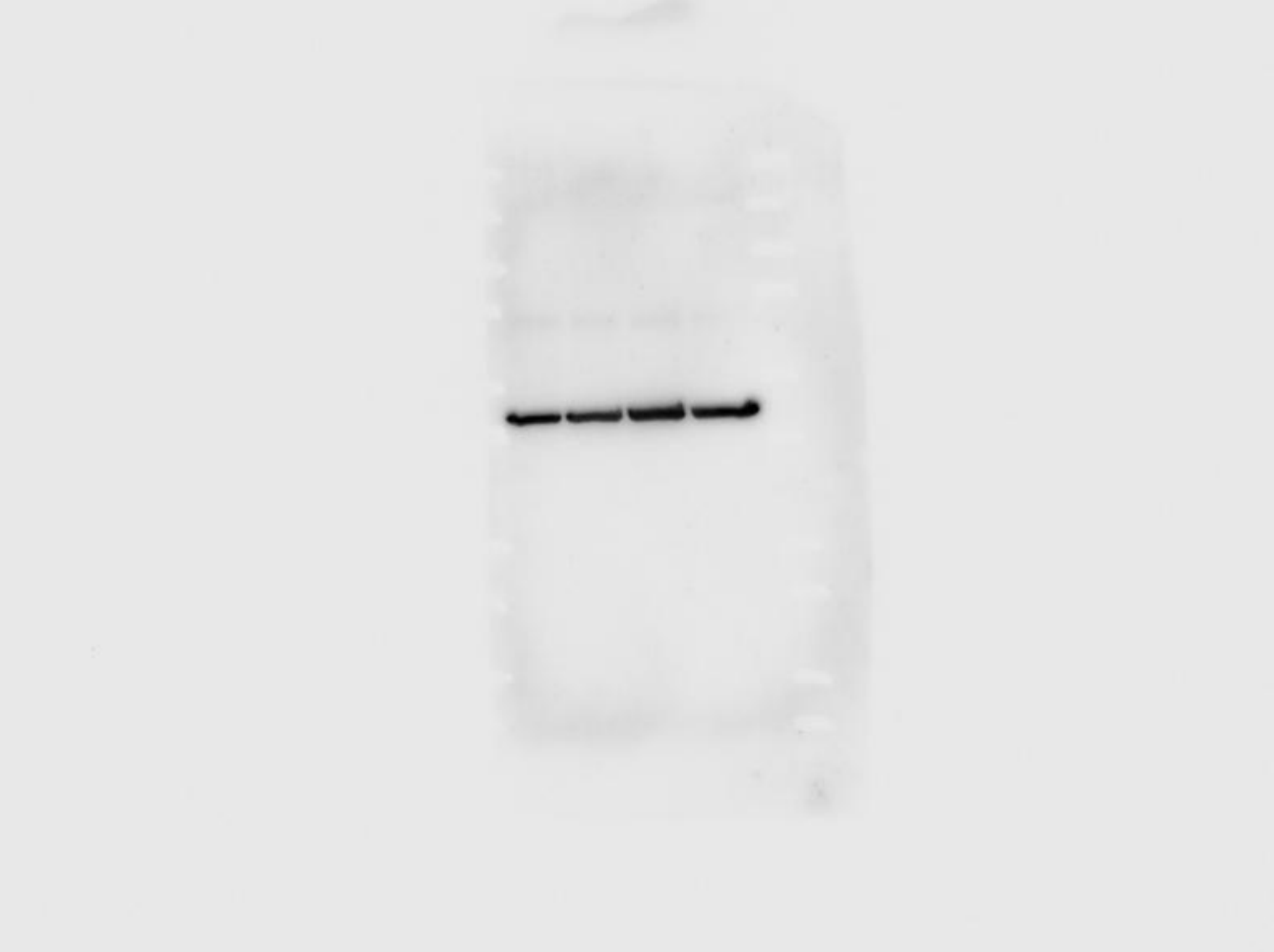

G

PPARγ

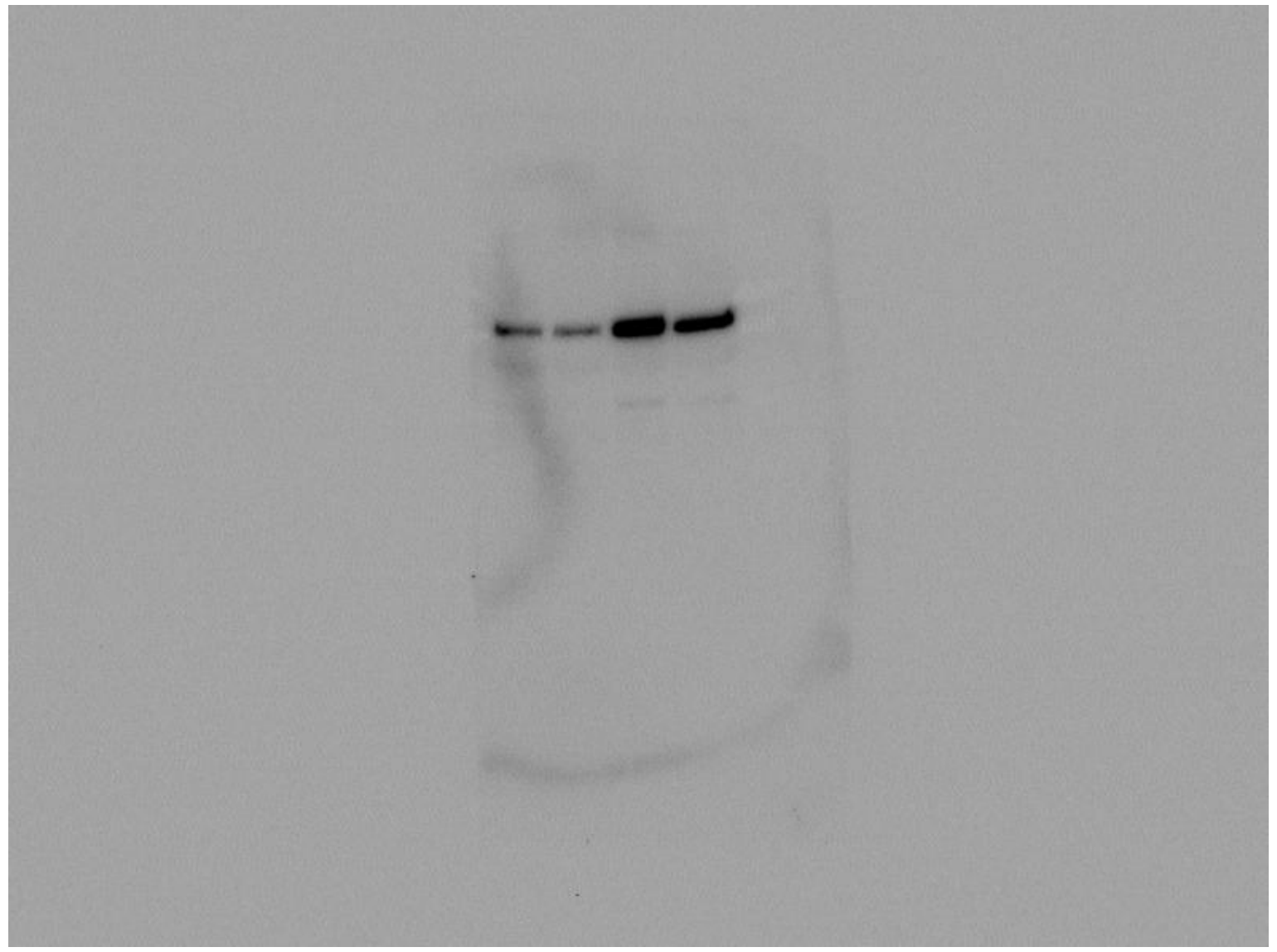

Supplement: Supplementary file 2 [file mmc3.pdf]
